# An *ELF4* hypomorphic variant results in NK cell deficiency

**DOI:** 10.1172/jci.insight.155481

**Published:** 2022-12-08

**Authors:** Sandra Andrea Salinas, Emily M. Mace, Matilde I. Conte, Chun Shik Park, Yu Li, Joshua I. Rosario-Sepulveda, Sanjana Mahapatra, Emily K. Moore, Evelyn R. Hernandez, Ivan K. Chinn, Abigail E. Reed, Barclay J. Lee, Alexander Frumovitz, Richard A. Gibbs, Jennifer E. Posey, Lisa R. Forbes Satter, Akaluck Thatayatikom, Eric J. Allenspach, Theodore G. Wensel, James R. Lupski, H. Daniel Lacorazza, Jordan S. Orange

**Affiliations:** 1 Department of Pediatrics, Baylor College of Medicine, Texas Children’s Hospital, Houston, Texas, USA.; 2 Department of Pediatrics, Columbia University Irving Medical Center, New York, New York, USA.; 3 Department of Pathology & Immunology,; 4 Department of Biochemistry,; 5 Department of Molecular and Human Genetics, and; 6 Human Genome Sequencing Center, Baylor College of Medicine, Houston, Texas, USA.; 7 Division of Pediatric Allergy, Immunology, and Rheumatology, Department of Pediatrics, University of Florida, Shands Children’s Hospital, Gainesville, Florida, USA.; 8 Division of Immunology, Seattle Children’s Hospital, Seattle, Washington, USA.; 9 Department of Pediatrics, University of Washington, Seattle, Washington, USA.

**Keywords:** Cell Biology, Immunology, Innate immunity, Monogenic diseases, NK cells

## Abstract

NK cell deficiencies (NKD) are a type of primary immune deficiency in which the major immunologic abnormality affects NK cell number, maturity, or function. Since NK cells contribute to immune defense against virally infected cells, patients with NKD experience higher susceptibility to chronic, recurrent, and fatal viral infections. An individual with recurrent viral infections and mild hypogammaglobulinemia was identified to have an X-linked damaging variant in the transcription factor gene *ELF4*. The variant does not decrease expression but disrupts ELF4 protein interactions and DNA binding, reducing transcriptional activation of target genes and selectively impairing ELF4 function. Corroborating previous murine models of ELF4 deficiency (*Elf4^–/–^*) and using a knockdown human NK cell line, we determined that ELF4 is necessary for normal NK cell development, terminal maturation, and function. Through characterization of the NK cells of the proband, expression of the proband’s variant in *Elf4^–/–^* mouse hematopoietic precursor cells, and a human in vitro NK cell maturation model, we established this *ELF4* variant as a potentially novel cause of NKD.

## Graphical abstract

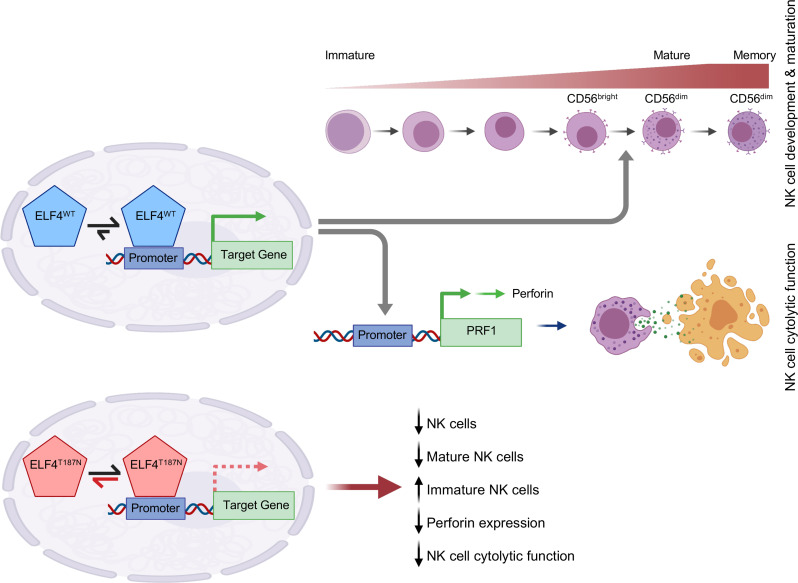

## Introduction

Primary immunodeficiencies (PID) are inborn errors of immunity (IEI) in which genetic variants affect the immune system (1). Understanding the pathogenesis of gene variants provides mechanistic insights into immune functions and immunological therapies. Clinically, IEI present as susceptibility to infections or immune dysregulation. NK cell deficiencies (NKD) represent PID in which the major immunological defect is within NK cells (1). NKD are subtyped as functional NKD, in which mature NK cells are present but functionally impaired, or classical NKD (cNKD), in which NK cell development/maturation and function are impaired (1). There are more than 450 IEI genes, with 7 known to cause NKD, each providing novel biological insights (1–4).

NK cells are critical in the innate response against viral infections and malignancy and help shape immune responses (1, 5). They can mediate cytotoxicity by releasing cytolytic proteins and activate/regulate immunity by releasing interferon-γ (IFN-γ) and other cytokines (5). Human NK cells are CD3^–^CD56^+^ and have 2 major subsets in peripheral blood: CD56^bright^ and CD56^dim^. The former are considered less mature and immunoregulatory, while the latter represent approximately 90% and are effective in mediating cytotoxicity (6), although plasticity has been demonstrated (7, 8).

NK cell development begins in the bone marrow and continues within secondary lymphoid organs (5, 9). In human NK cells, 6 developmental stages have been identified with 2 of them bifurcated (9): stage 1: CD34^+^CD117^–^, stage 2: CD127^+^CD34^+^CD117^+^ (stage 2a: IL1R1^–^, stage 2b: IL1R1^+^), stage 3: CD127^–^CD34^–^CD117^+^CD56^–^, stage 4: CD34^–^CD117^+/–^CD56^+^CD16^–^ (stage 4a: NKp80^–^, stage 4b: NKp80^+^), stage 5: CD117^–^CD56^+^CD16^+^CD57^–^, and stage 6: CD56^+^CD16^+^CD57^+^ (5). In mice, NK cell maturation is defined by the surface expression of CD27 and CD11b (5, 10), which occurs consecutively: CD27^+^CD11b^–^→CD27^+^CD11b^+^→CD27^–^CD11b^+^, referred to as immature, mature, and terminally mature, respectively (5, 11).

NK cell development is additionally characterized by differential expression of key transcription factors (12). The E-twenty-six (Ets) winged helix-turn-helix proteins share an Ets domain that binds purine-rich DNA (13), which target motifs enriched in lymphocyte-specific gene loci (14) and function to promote proliferation, lineage differentiation, and apoptosis (13). ELF4 is an Ets family member with important regulatory targets in NK cells, including perforin and genes that regulate NK cell development. *Elf4*^−/−^ mice have decreased NK cell–specific cytotoxic function and perforin expression, which IL-2 fails to rescue (15). Additionally, murine Elf4 deficiency impairs NKT and NK cells, but not T cell development, suggesting that Elf4 is preferentially required by NK cells, although the specific Elf4-dependent stage is unclear (15). The role of *ELF4* in human NK cell development is also unclear. Here we have characterized roles of ELF4 in human NK cell development and function and identified a specific patient-derived hypomorphism of *ELF4* that can cause an NKD.

## Results

### Identification of an individual with a potential NKD.

**Figure 1:**
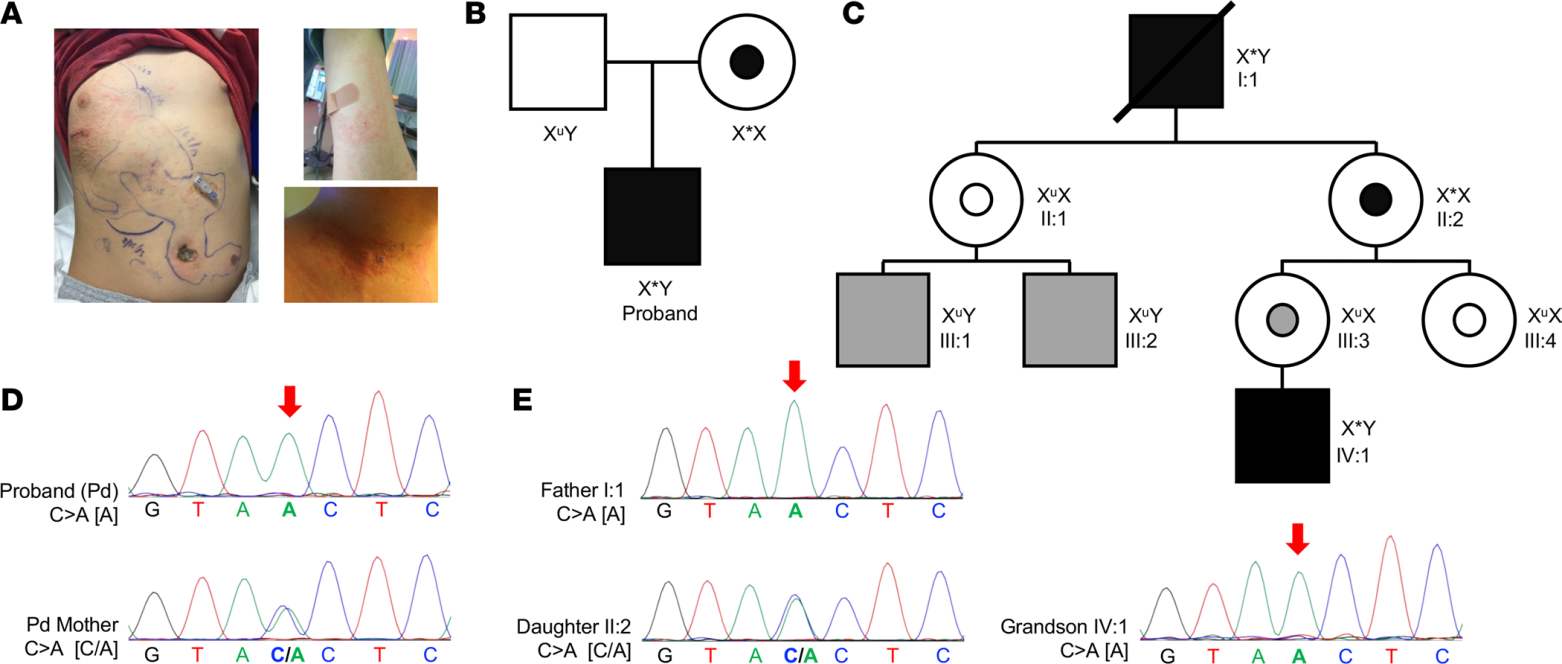
Clinical phenotype and genotype of an individual with presumed NKD. (**A**) Persistent herpes zoster lesions on proband crossing dermatomes showing trunk (left), arm, and neck (right). (**B**) Pedigree of the proband with X-linked inheritance. (**C**) Pedigree of a second family with *ELF4* c.560C>A variant. Pedigrees show affected (black), carrier (black dot), unknown carrier (white dot), predicted carrier (gray dot), unknown (gray), and unaffected (white) individuals with their respective genotype: X* (c.560C>A *ELF4* confirmed), X^u^ (unconfirmed genotype), and X (presumably wild-type *ELF4*). (**D**) Sanger sequencing of *ELF4* in proband and his mother (arrow indicates nucleotide change c.560C>A. (**E**) Sanger sequencing of the genotyped individuals in the second family.

An autistic male child with a history of recurrent lower respiratory tract and ear infections requiring tympanostomy tube placement, tonsillectomy, and adenoidectomy (Supplemental Table 1; supplemental material available online with this article; https://doi.org/10.1172/jci.insight.155481DS1) was diagnosed with mild hypogammaglobulinemia (IgG 584 mg/dL, IgA 85 mg/dL, IgM 54 mg/dL, IgE 12 kU/L) and treated with immunoglobulin replacement. Despite receiving the varicella-zoster virus vaccine on schedule, he developed zoster at age 9. He continued to have recurrent (>10) outbreaks that crossed dermatomes (Figure 1A) that responded to valacyclovir. T and B lymphocyte subsets were within normal ranges (Table 1), though he had a 1:1 CD4/CD8 ratio with an appropriate distribution of RA^+^/RO^+^ CD4^+^ T cells (Supplemental Table 2). Lymphocyte mitogen and antigen proliferative responses were normal, as was the distribution of B cell subsets except switched memory B cells, which were low (16). NK cells, however, were consistently reduced in frequency and absolute number (Table 1). Given the atypical recurrent herpes zoster, intact T cell numbers and function, and absence of other significant uncorrected immunological abnormalities, an NKD was considered.

Exome sequencing was performed to identify a gene variant potentially associated with the clinical phenotype (Supplemental Table 3). A bioinformatically significant rare hemizygous nonsynonymous variant, in the transcription factor gene *ELF4* (ChrX:129205367_G>T[GRCh37/hg19]:NM_001421:exon 6:c.560C560C>A;p.T187N) having a hemizygous frequency of 0.00006241 in the gnomAD database_v3.1.2 (17) predicting a missense alteration was identified. An X-linked recessive trait where the mother is a heterozygous carrier was predicted (Figure 1B) and confirmed by Sanger sequencing (Figure 1D).

The *ELF4* c.560C>A variant identified in the proband resides in an evolutionarily conserved region and encodes an amino acid change in ELF4 (p.T187N), with a Combined Annotation-Dependent Depletion (18) score of 22.6, mutation significance cutoff of 3.313, and PolyPhen2 (19) and likelihood ratio statistical test predicting deleterious impact without affecting protein stability (Supplemental Table 3).

ELF4 is highly expressed in immune cells and known to transactivate hematopoietic and lymphocyte-relevant gene promoters, and murine models of ELF4 deficiency (*Elf4^–/–^* mice) have diminished NK cells having decreased cytolytic function (15). Given the bioinformatic significance of the variant combined with the biological relevance of *ELF4* to NK cells, we considered it further as a potentially novel NKD gene candidate.

Subsequently, a second family was independently identified to carry the same c.560C>A p.T187N *ELF4* variant (Figure 1C). A female patient initially having recurrent sinusitis and later lymphoproliferation was diagnosed with common variable immunodeficiency (CVID). The *ELF4* variant in this individual was identified via familial exome sequencing, which also identified the hemizygous individuals, the now-deceased father and male descendant (Figure 1E). The affected male individual/father had a history of recurrent oral ulcers and severe infections since childhood. In his 50s, he developed recurrent malignancies, including lymphomatoid papulosis, recurrent angioimmunoblastic T cell lymphoma, and 2 distinct cutaneous CD30^+^ anaplastic large T cell lymphomas (Supplemental Table 1). Immunophenotyping at age 75 (I:1) was performed and presented alongside that of his daughter (II:2) (Supplemental Table 2). Individual II:2 had hypogammaglobulinemia (IgG 15 mg/dL, IgA 2 mg/dL, undetectable IgM and IgE), mild lymphopenia (Supplemental Table 1), and decreased NK cells (Table 1). The etiology of her CVID remains under investigation. Her father (I:1) had mild leukopenia, and while his lymphocyte subset analysis did not include NK cells (Table 1), his B cells were decreased, as were IgG and IgA class-switched memory B cells and immature/transitional B cells, but levels of IgG, IgM, and IgA were normal (Table 1 and Supplemental Table 1). Unfortunately, apart from one set of values collected during active hematologic malignancy, assessments of NK cells were not part of his evaluations. Further study was not possible owing to his death; however, his clinical history indicates concerns, including cancer susceptibility. Due to limited access and the young age of the male descendant, individual IV:1, it is unclear if he is affected; at this time, there are no concerns reported by the family.

### Characterization of NK cell phenotype in an individual with ELF4 T817N.

**Figure 2:**
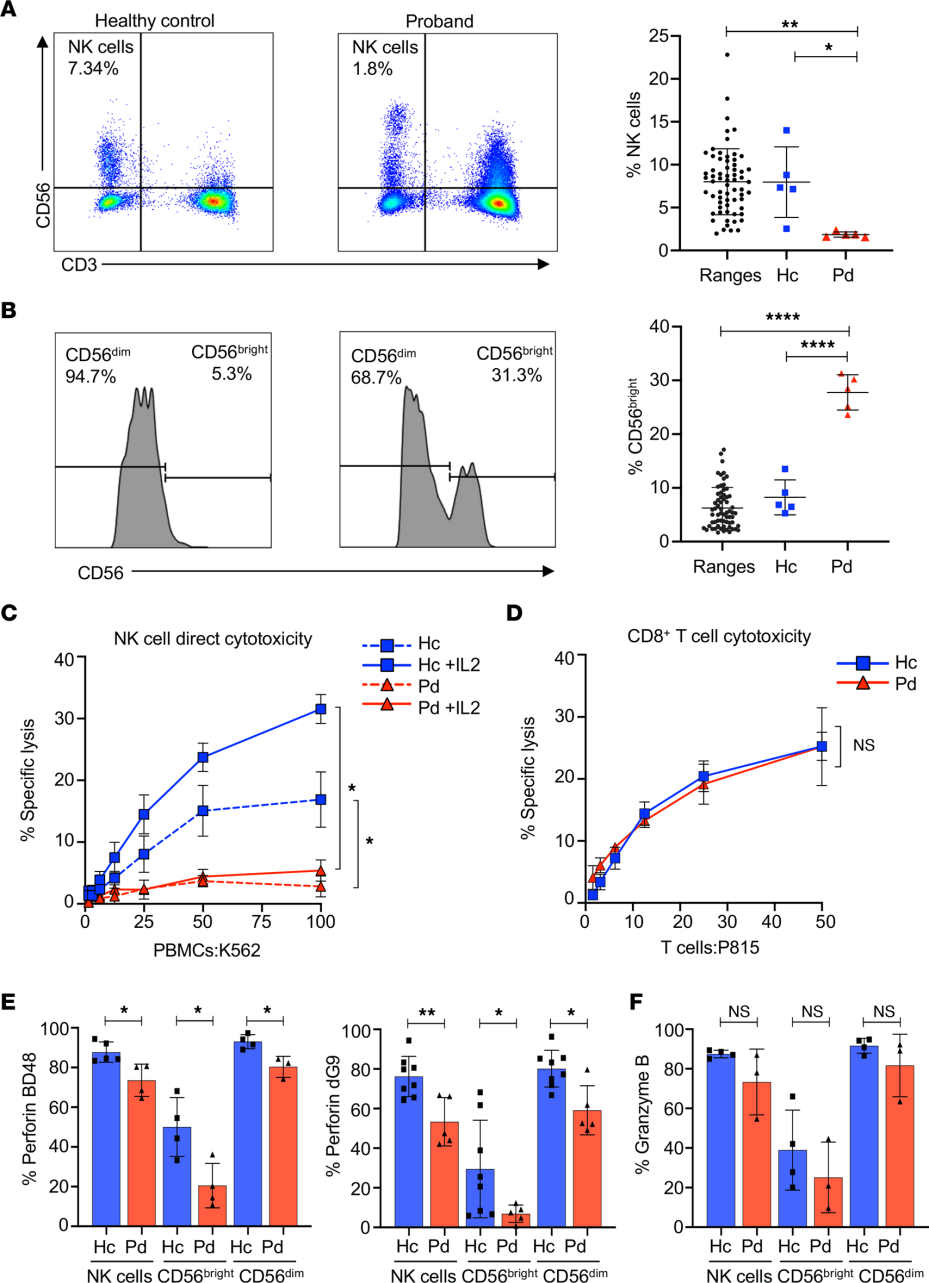
Reduced NK cell frequency is accompanied by impaired NK cell function and decreased perforin expression in the proband. PBMCs from proband (red) and healthy control (blue) were used to assess NK cell phenotype. (**A**) NK cell frequency in the proband compared with healthy control and previously determined normal ranges (20) with FACS plots of NK cells defined as CD56^+^CD3^–^. (**B**) CD56^bright^ NK cell frequency with histograms delineating CD56^bright^ and CD56^dim^ in the proband and healthy control (left) and compared with previously defined normal ranges and healthy control (right). (**C**) ^51^Cr release assay using PBMCs against K562 target cells with (solid line) and without (dashed line) IL-2 stimulation cell-mediated direct NK cell cytotoxicity. (**D**) CD8^+^ T cell cytotoxicity assay using in vitro expanded cytotoxic T lymphocytes against P815 target cells preincubated with anti-CD3. (**E**) Frequency of nascent (left) and processed (right) perforin in total NK cells and CD56^bright^/CD56^dim^ subsets using perforin antibody clones BD48 and δG9, respectively. (**F**) Frequency of granzyme B in total NK cells and CD56^bright^/CD56^dim^ subsets. Data represent mean ± SEM of ≥3 biological replicates (4 for NK cell cytotoxicity and 3 for T cell cytotoxicity, *n* ≥ 4 for all other); *P < 0.05, **P < 0.01, ****P < 0.0001; 2-tailed Student’s *t* test with Bonferroni’s correction for multiple comparisons and Wilcoxon matched pairs signed-rank test for specific lysis curves.

Analysis of the NK cell phenotype in the living proband of the first case was performed using peripheral blood. The percentage of CD3^–^CD56^+^NK cells were further delineated as CD56^bright^ and CD56^dim^ NK cell subsets (20), as the former is considered immature, despite serving as potent cytokine producers with an immunoregulatory role (21), while the latter is mature and predominantly cytotoxic (1). Upon multiple evaluations, the proband had reduced percentages of NK cells compared with controls and previously established population ranges (20) (Figure 2A). The proband also had a significant increase in the immature CD56^bright^ NK cell population compared with controls and previously defined normal ranges (20) (Figure 2B). Although individual II:2 had decreased NK cells, normal CD56^bright^/CD56^dim^ NK cell ratios were demonstrated (Supplemental Table 1, data not shown). Thus, the proband had a consistent, stable reduction in NK cells in peripheral blood with increased representation of the immature CD56^bright^ NK cell subset.

Next, we evaluated the proband’s NK cell lytic function via direct and antibody-dependent cellular cytotoxicity (ADCC) using PBMCs. Throughout 6 years, the proband had decreased direct NK cell cytolytic function that remained so after in vitro stimulation with IL-2, as well as reduced ADCC (Figure 2C and Supplemental Figure 1A). The deficiency in NK cell cytotoxicity was also apparent when the activity was expressed as lytic units (LU) per PBMC (Supplemental Figure 1B). Given that the proband had low NK cell percentages, we normalized to the percentage of NK cells subsequently to CD56^dim^ NK cells, defining the LU per NK cell and LU per CD56^dim^ NK cell. The significant difference between the control and the proband after normalization only remained for IL-2–stimulated killing (Supplemental Figure 1, C and D). Thus, the proband portrays impaired ex vivo NK cell cytotoxicity that is partly due to decreased CD56^dim^ NK cell frequency but is more pronounced per NK cell when stimulated with IL-2. However, evaluation of expanded proband T cells demonstrated no defect in their lytic function (Figure 2D), suggesting the cytolytic deficiency was NK cell specific.

Previous studies of *Elf4^–/–^* mice identified decreased frequencies of NK cells, reflecting results of our proband, and further demonstrated that Elf4 transactivates the perforin promoter and is required for constitutive perforin expression in NK cells (15). Thus, we next evaluated the expression of NK cell cytolytic proteins, granzyme B, and both processed and nascent perforin (using antibody clones δG9 and BD48, respectively) (20, 22). We found a significant decrease in perforin but not granzyme B–positive NK cell frequency within the proband’s total NK cell population within the CD56^dim^ and CD56^bright^ subsets compared to control and previously reported ranges (Figure 2, E and F, and data not shown) (20). Analysis of stimulated CD8^+^ T cells also demonstrated decreased perforin but normal total granzyme B expression (Supplemental Figure 1E), although they again had normal lytic function (unlike the NK cells).

### Mice expressing ELF4^T187N^ yield decreased NK cells with reduced maturity and function.

**Figure 3:**
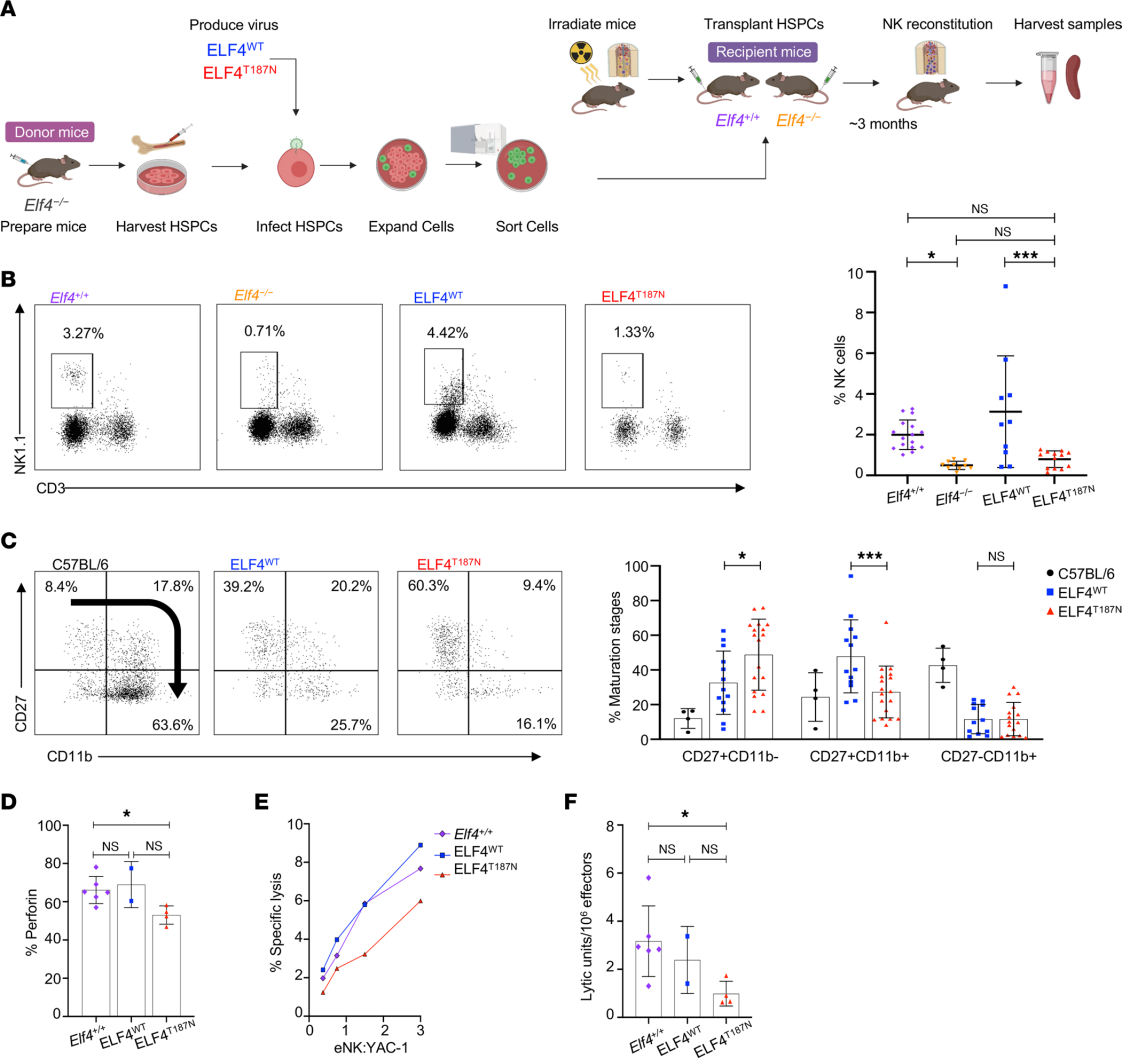
Reduced NK cell frequency is accompanied by impaired NK cell function and decreased perforin expression in a murine ELF4 BM chimera model. (**A**) ELF4 BM chimera model experimental design. ELF4 wild-type or T187N-IRES-GFP constructs were expressed in C57BL/6 *Elf4^–/–^* HSPCs and transplanted into recipient *Elf4*^+/+^ or *Elf4^–/–^* mice. (**B**) Percentage of ectopic expression of ELF4^WT^ (blue) and ELF4^T187N^ (red) and endogenous expression of *Elf4^+/+^* (purple) or *Elf4^–/–^* (orange) from blood samples in murine NK cells identified as CD3^–^NK1.1^+^. (**C**) NK cell maturation subsets from spleen samples identified by their CD11b and CD27 expression maturing as indicated by the arrow. Comparing control mice (black) and NK cells expressing the ELF4^WT^ (blue) and ELF4^T187N^ (red) constructs. (**D**) Perforin-positive splenic NK cells from ELF4^WT^, ELF4^T187N^, and *Elf4^+/+^* control samples from polyI:C-stimulated mice. (**E**) NK cell cytotoxicity against YAC-1 target cells measured via Cr^51^ release assay using sorted NK cells expressing *Elf4^+/+^* control, ELF4^WT^, and ELF4^T187N^ from the spleens of polyI:C-stimulated chimeric mice. (*n* = 6: *Elf4^+/+^*, 2: ELF4^WT^, and 4: ELF4^T187N^.) (**F**) LU calculated from the previous cytotoxicity assay. The data represent mean ± SEM of 3 independent experiments; each symbol represents an individual mouse with an *n* of ≥4 unless otherwise stated; *P < 0.05, ***P < 0.001; 2-tailed Student’s *t* test with Bonferroni’s correction for multiple comparisons.

*Elf4*^−/−^ mice do not have embryonic lethality, developmental abnormalities, or pervasive immune aberrations (15). In mice, *Elf4* regulates hematopoietic stem and progenitor cell (HSPC) quiescence during steady-state hematopoiesis (23), inhibits proliferation of naive CD8^+^ T cells (24), and regulates the development and function of NK cells (15). *Elf4*^−/−^ mice housed in specific pathogen–free (SPF) conditions have quantitatively decreased NK cells, which also have decreased cytolytic function (15), a phenotype similar to that of the proband expressing the ELF4 p.T187N variant. To better understand the impact of the ELF4 p.T187N variant on NK cell development and function, we employed a bone marrow chimera model (11). We performed transduction of *Elf4^–/–^* C57BL/6 donor HSPCs with retrovirus carrying *ELF4^WT^* or *ELF4^T187N^* IRES GFP constructs (Figure 3A). After expansion and cell sorting, GFP^+^ HSPCs were transplanted into either *Elf4^+/+^* or *Elf4^–/–^* recipient irradiated mice. Three months were allowed for NK cell reconstitution, which was confirmed before collecting the final blood and spleen samples. This approach yielded 4 experimental mouse groups: *Elf4*^+/+^ or *Elf4*^–/–^ recipients, each with ectopic expression of ELF4^WT^ or ELF4^T187N^.

We analyzed blood and spleen samples from the bone marrow chimera mouse model using flow cytometry, identifying ELF4^WT^- or ELF4^T187N^-derived cells by GFP expression and then gating NK cells from within the lymphocyte population by CD3^–^NK1.1^+^ expression (5). As previously described, the absence of Elf4 expression (*Elf4^–/–^*) led to a significant decrease of NK cells in peripheral blood and lymphoid tissues (15); however, ectopic expression of wild-type ELF4 (ELF4^WT^) protein in *Elf4^–/–^* HSPCs normalized the NK cell frequency in peripheral blood to that of ELF4^+/+^ mice (Figure 3B). Notably, the ectopic expression of the ELF4 p.T187N variant (ELF4^T187N^) led to a decrease in the frequency of NK cells similar to the phenotype observed in untransduced *Elf4^–/–^* mice (Figure 3B). When evaluated across multiple mice and independent experiments, the ectopic expression of ELF4^T187N^, when compared with the ectopic expression of its ELF4^WT^ counterpart, resulted in a significant decrease in the percentage of NK cells in peripheral blood (Figure 3B). Thus, the ELF4 p.T187N variant results in an NK cell–intrinsic defect, decreasing the frequency of NK cells similar to that observed in Elf4-deficient mice and reflecting values observed in the proband.

Given the decreased frequency of mature NK cells in the proband and the decreased peripheral blood NK cells in our mouse model, we evaluated NK cell maturation in samples from each group of mice. Despite the previously reported decrease in NK cell numbers in *Elf4^–/–^* mice, the impact on NK cell developmental intermediates (NKDIs) had not been determined. Through our model, we found that *Elf4^–/–^* splenic NK cells had significantly increased CD27^+^CD11b^–^ immature and decreased CD27^–^CD11b^+^ mature NK cells compared with *Elf4^+/+^* controls (Supplemental Figure 2). While experimental mice with ectopic ELF4^WT^ and ELF4^T187N^ from both conditions had decreased CD27^–^CD11b^+^ maturation compared with the C57BL/6 unmodified control mice, no significant difference existed in the frequency of CD27^–^CD11b^+^ cells between them (Figure 3C). That said, we found a significantly decreased intermediate mature stage CD27^+^CD11b^+^ and an increased percentage of immature CD27^+^CD11b^–^ in the NK cells expressing ELF4^T187N^ compared with the ELF4^WT^ (Figure 3C). The CD27^+^CD11b^–^ immature population in NK cells expressing ELF4^T187N^ was substantially higher than that found in C57BL/6 mice and comparable to that found in *Elf4^–/–^* mouse spleens. Although it is difficult to directly compare between the increased CD56^bright^ immature NK cell phenotype of the proband and the mouse model, it is notable that mouse samples expressing the p.T187N ELF4 variant mirror the phenotype of having more immature NK cells.

Given the role Elf4 plays in transactivating the perforin promoter, presumably independently of a role in NK cell development (15) and the proband’s decreased perforin-positive NK cells, we analyzed perforin expression in our chimeric mouse model. Since mice in SPF facilities have little baseline perforin expression, we attempted to induce expression by 100 μg polyI:C treatment 24 hours prior to sample collection. We observed a similar frequency of perforin-positive NK cells in the *Elf4^+/+^* control and ectopic ELF4^WT^ samples and a significant decrease in those ectopically expressing ELF4^T187N^ (Figure 3D). While the expression of perforin was severely impaired in *Elf4^–/–^* mice (15), some expression was observed in ELF4^T187N^ splenic NK cells, suggesting reduced transactivation of the perforin promoter leading to suboptimal, but not absent, protein expression. Thus, the T187N variant in ELF4 results in decreased perforin-positive splenic NK cells similar to that observed in the peripheral blood NK cells of the proband.

Finally, we evaluated NK cell cytolytic activity in our mice. Since spontaneous NK cell cytotoxicity is negligible in mice housed under SPF conditions, we used polyI:C-stimulated splenic NK cells from chimeric mice as effectors against YAC-1 susceptible target cells in an ex vivo ^51^Cr cytotoxicity assay. Lysis of YAC-1 target cells was decreased in the ELF4^T187N^ cells relative to ELF4^WT^ and *Elf4^+/+^* (Figure 3E). To compare across independently repeated experiments and mice better, the LU per million effector NK cells required to kill 5% of the target cells were calculated, demonstrating decreased LU of the ELF4^T187N^ NK cells compared with ELF4^WT^ and significantly decreased LU compared with *Elf4^+/+^* NK cells (Figure 3F). Thus, expression of ELF4^T187N^ resulted in decreased cytotoxicity associated with reduced frequency of perforin-positive NK cells. The *Elf4^–/–^* mice housed in SPF conditions did not present immune abnormalities other than NK cell defects and did not present overt signs of inflammation such as anorexia/cachexia. Similarly, the chimeric mouse model demonstrated that the expression of ELF4^T187N^ leads to an intrinsic NK cell abnormality with decreased NK cell percentage, increased immaturity, diminished perforin expression, and reduced function, similar to what was observed in the proband.

### ELF4^T187N^ HSPCs give rise to incompletely matured NK cells.

**Figure 4:**
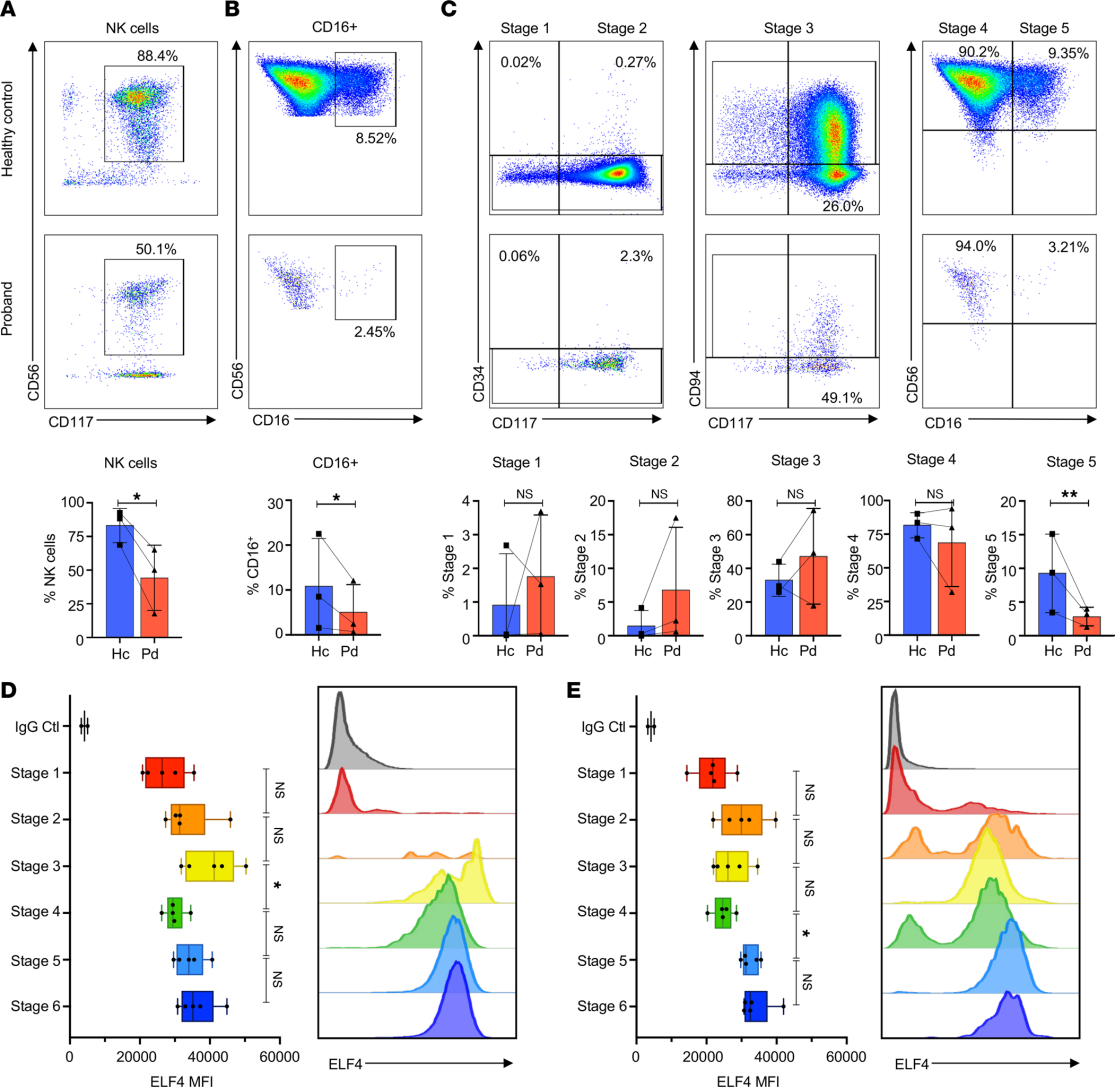
Expression of ELF4 in human NK cell precursors reflects its role in maturation. (**A**–**C**) Isolated CD34^+^ precursors were cultured with stromal cells for 4 weeks to generate mature NK cells in vitro, with the lines in the bar graphs (bottom) connecting experimental repeats. (**A**) NK cell frequency from healthy control and proband samples. (**B**) Frequency of the mature population of the NK cell gate identified as CD16^+^. (**C**) Additional analysis of the same in vitro differentiation data (as shown in **A** and **B**) identifying the frequency of the NK cell developmental stages as Lin^–^ stage 1 CD34^+^CD117^–^, stage 2 CD34^+^CD117^+^, stage 3 CD34^–^CD117^+^CD94^–^, stage 4 CD34^–^CD117^+^CD94^+^CD56^+^CD16^–^, and stage 5 CD34^–^CD117^+^CD94^+^CD56^+^CD16^+^. (**D** and **E**) ELF4 expression analyzed by flow cytometry in NK cell precursors from human peripheral blood (**D**) or tonsil (**E**) analyzed as per Supplemental Figure 3A. ELF4 MFI of the positive cells with representative histograms. (*n* = 5.) Box plots show the interquartile range (box), median (line), and minimum and maximum (whiskers). Data represent mean ± SEM of ≥3 biological replicates unless *N* otherwise specified; *P < 0.05, **P < 0.01, 2-tailed Student’s *t* test (**A**–**C**) and ANOVA with multiple comparisons (**D** and **E**).

Previous studies have demonstrated that ELF4 is necessary for NK cell lineage commitment (15); however, a role for *ELF4* in human NK cell development has not been described to our knowledge. The effect of the ELF4 p.T187N variant on human NK cell development was evaluated by an in vitro NK cell differentiation assay from CD34^+^ precursors isolated from the peripheral blood of the proband or healthy donors. Specifically, isolated CD34^+^ cells were cocultured with irradiated EL08.1D2 stromal cells in the presence of cytokines, as previously described (3). After 4 weeks, we detected a significant reduction in the number and frequency of NK cells, defined as CD34^–^CD56^+^ (Supplemental Figure 3A), in the proband sample compared with controls (Figure 4A). Of the total NK cells identified in the cultures, we also observed a significant decrease in the frequency of mature CD16^+^ NK cells in the proband sample relative to controls (Figure 4B). As we only evaluated the cells from our assay at 4 weeks (Supplemental Figure 3A), very few early precursors remained at this time point even in control conditions, and we observed no consistent change from the control in the small percentages of stage 1–3 precursors in the proband (Figure 4C). We found no significant difference in the relative frequency of stage 4 NK cells generated, whereas we found a significant decrease in the frequency of stage 5 (CD56^+^CD16^+^) NK cells derived from the proband CD34^+^ cells (Figure 4C). Thus, the presence of ELF4 p.T187N potentially impacts NK cell development, particularly in the maturation of stage4 CD56^bright^CD16^–^ to stage 5 CD56^dim^CD16^+^ NK cells, suggesting that ELF4 functions in human NK cell maturation.

### ELF4 expression correlates with human NK cell maturation.

As relative ELF4 expression levels correlate with its function (25), we sought to define the expression of ELF4 in human NK cell precursors. A 13-color flow cytometry panel and gating strategy was used to delineate NKDIs (26, 27) and detect ELF4 within peripheral blood and tonsils from healthy individuals (Supplemental Figure 3B). In both blood and tonsils, only a small percentage of stage 1 cells were ELF4^+^, whereas most stage 2–6 cells were ELF4^+^ (Supplemental Figure 3, C and D, respectively). ELF4 mean fluorescence intensity (MFI) measured in peripheral blood NK cell subsets and precursors trended toward increased expression as cells matured (Figure 4D). Early NKDIs from peripheral blood are infrequent; thus, the small number of stage 3 cells present, which had the highest ELF4 expression levels (Figure 4D), could have innate lymphoid cells and precursors within despite our efforts to exclude them via gating. Tonsil is a known secondary lymphoid tissue supporting NK cell maturation (28, 29), giving the increasing expression of ELF4 with increasing NK cell maturity more substantial implications. The significant increase from stage 4 to stage 5 (Figure 4E), further delineating stage 4 via NKp80, separating stage 4a (NKp80^–^) from stage 4b (NKp80^+^) (Supplemental Figure 4A), indicates commitment to maturity (9, 27). ELF4 expression increased between stages 4a, 4b, and 5 (changes significant in tonsillar NK cells, Supplemental Figure 4, B and C). Analysis of Ki67^+^ proliferating cells identified significance in the frequency of Ki67^+^ cells correlating with ELF4 expression in stages 4 and 5 in the tonsil (Supplemental Figure 5). Thus, while ELF4 expression generally follows NK cell maturation, significant increases from stage 4 to 5 combined with the proband’s NK cell immature phenotype suggest that ELF4 contributes to terminal NK cell maturation.

### ELF4 regulates perforin expression in human NK cells.

**Figure 5:**
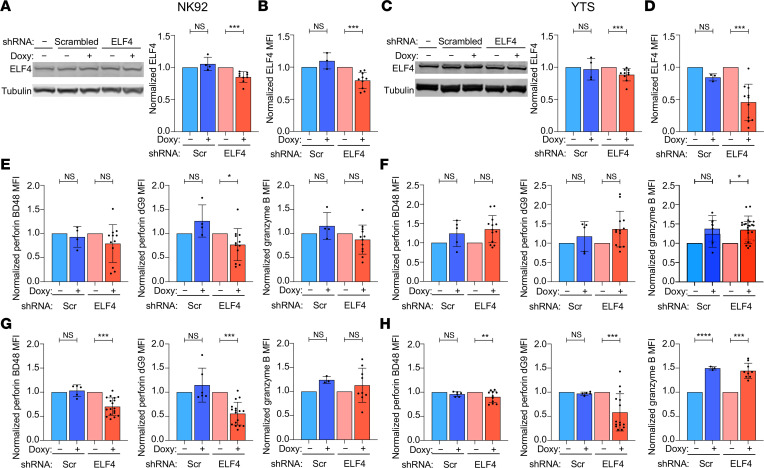
ELF4 regulates perforin expression in human NK cell lines. Doxycycline-inducible ELF4 shRNA or scramble control were expressed in NK92 and YTS human NK cell lines. (**A** and **C**) Representative Western blot (left) of samples without or with doxycycline treatment and parental cells with graph of ELF4 expression normalized to loading control and subsequently normalized to respective doxycycline-treated negative control (right) in NK92 (**A**) and YTS (**C**) cells. (**B** and **D**) ELF4 expression (MFI) measured by flow cytometry and normalized to respective doxycycline-treated negative control in NK92 (**B**) and YTS (**D**) cells. (**E** and **F**) Nascent (left) and processed (middle) perforin and granzyme B (right) MFI measured by FACS and normalized to respective doxycycline-treated negative control in NK92 (**E**) and YTS (**F**). (**G** and **H**) 48 hours after treatment with CMA, nascent (left) and processed (middle) perforin and granzyme B (right) MFI normalized to respective doxycycline-treated negative control in NK92 (**G**) and YTS (**H**) cells. Data represent mean ± SEM ≥3 biological replicates (*n* ≥ 4 scr, ≥9 ELF4); *P < 0.05, **P < 0.01, ***P < 0.001, ****P < 0.0001, 2-tailed Student’s *t* test.

Although our proband and mouse model demonstrated decreased NK cell counts, maturity, and perforin content, we wanted to determine if ELF4 has a direct role in perforin production independent of its role in NK cell maturation. Using human NK cell lines, NK92 and YTS, modest knockdown (KD) of ELF4 expression was achieved using a doxycycline-inducible shRNA system, which was validated by measuring ELF4 protein expression by Western blot and intracellular flow cytometry (Figure 5, A–D). Nascent perforin, measured with antibody clone BD48; processed perforin, measured with antibody clone δG9 (22); and granzyme B were assessed after ELF4 knockdown. No significant reduction in nascent perforin or granzyme B expression was identified after doxycycline treatment, but processed perforin was decreased in the NK92 cells (Figure 5, E and F). Because these cells had baseline expression of cytolytic proteins before ELF4 knockdown, we attempted to remove the preexisting proteins by deacidifying lytic granules with concanamycin A (CMA), which degrades perforin and granzymes. Two days after CMA treatment, the content of cytolytic proteins when measured by flow cytometry identified a significant decrease in the intensity (MFI) of both nascent and processed perforin but not granzyme B in both doxycycline-treated YTS (Figure 5G) and NK92 (Figure 5H). Thus, modest ELF4 KD in the clonal differentiated NK cell lines reduced perforin expression but not granzyme B, levels suggesting specificity for ELF4 in promoting perforin independently of NK cell maturation.

### ELF4 variant p.T187N maintains physiologic expression and localization.

**Figure 6:**
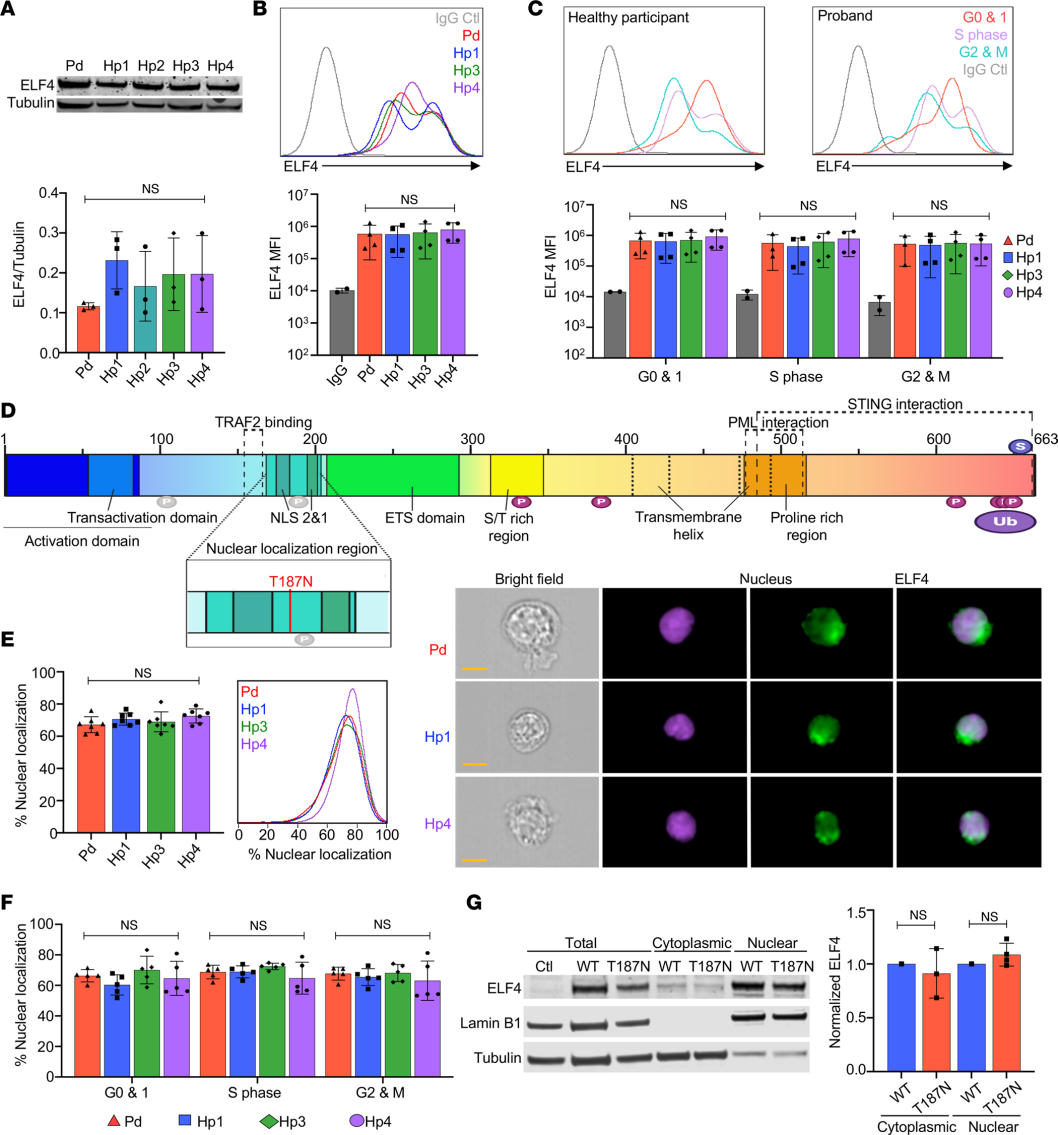
Normal ELF4 quantity and localization in the presence of c.C560A p.T187N. (**A**) Western blot of proband and healthy participant BLCLs (top) and quantification (bottom) of ELF4 expression normalized to tubulin loading control. (**B**) ELF4 MFI with a representative histogram (top) and quantification (bottom) assessed using imaging flow cytometry, with IgG control. (**C**) ELF4 MFI in different stages of cell cycle within representative histograms for healthy control and proband (top) and quantification (bottom), with IgG control. (**D**) ELF4 protein domains. P, phosphorylation sites predicted (gray) and experimentally confirmed (magenta); S, SUMOylation; Ub, ubiquitination; NLS, nuclear localization signal with T187N variant location (inset). (**E**) Proportion of ELF4 localizing to the nucleus in BLCLs assessed by imaging flow cytometry (left) with representative histograms (middle) and images (right) of bright-field, nucleus, and ELF4, and an overlay of ELF4/nuclear dye (scale bar: 7 μm). (**F**) Nuclear localization of ELF4 determined in different stages of the cell cycle. (**G**) Western blot of ELF4 wild-type and T187N variant overexpression in HEK293T in the nuclear and cytoplasmic fractions (left), with quantification (right), normalized to Lamin B1 and tubulin loading control for the respective fractions and further normalized to wild-type control. The data represent mean ± SEM of ≥3 independent experiments; images represent experiments repeated at least 3 times; 1-way ANOVA with multiple comparisons (**A**–**C**, **E**, and **F**), 2-tailed Student’s *t* test (**G**).

Given that the p.T187N ELF4 variant altered the biology of NK cells, we wanted to next determine mechanisms by which it affected ELF4 function (i.e., mutation impact testing). Using proband B lymphoblastoid cell line (BLCL), we first evaluated the variant’s effect upon ELF4 protein expression by Western blot (Figure 6A) and imaging flow cytometry (Figure 6B) and found no significant decrease. As ELF4 protein level in cell lines is controlled in a cell cycle–dependent manner (30, 31), we also measured ELF4 expression in G_0_/G_1_, S, and G_2_/M phases (notably, there was no abnormality in BLCL growth dynamics; data not shown). In each phase of the cell cycle, evaluated by imaging flow cytometry, the proband BLCLs expressed detectable levels of ELF4, comparable to controls (Figure 6C). Thus, the T187N variant did not appear to impact ELF4 protein expression levels.

Given the physiologic levels of ELF4 T187N, we next evaluated its subcellular localization. ELF4 contains 2 nuclear localization signals (30), and position 187 is located between them (Figure 6D). Using imaging flow cytometry, ELF4 nuclear levels in healthy donor– or proband-derived BLCLs demonstrated approximately 70% of ELF4 localized to the nucleus (Figure 6E). When gated at different cell cycle stages using BrdU and DAPI, there was also no significant difference in ELF4 nuclear localization corresponding to the cell cycle between proband and control (Figure 6F). To ensure the localization of ELF4 was not particular to BLCLs, we overexpressed WT or T187N ELF4 variants in HEK293T cells and performed cell fractionation and Western blotting (Figure 6G). The T187N variant and WT ELF4 were present in equivalent amounts in the cytoplasm and nucleus (Figure 6G), suggesting that the variant can attain normal expression levels and translocate into the nucleus.

### ELF4 variant p.T187N impairs protein function by decreasing DNA binding.

**Figure 7:**
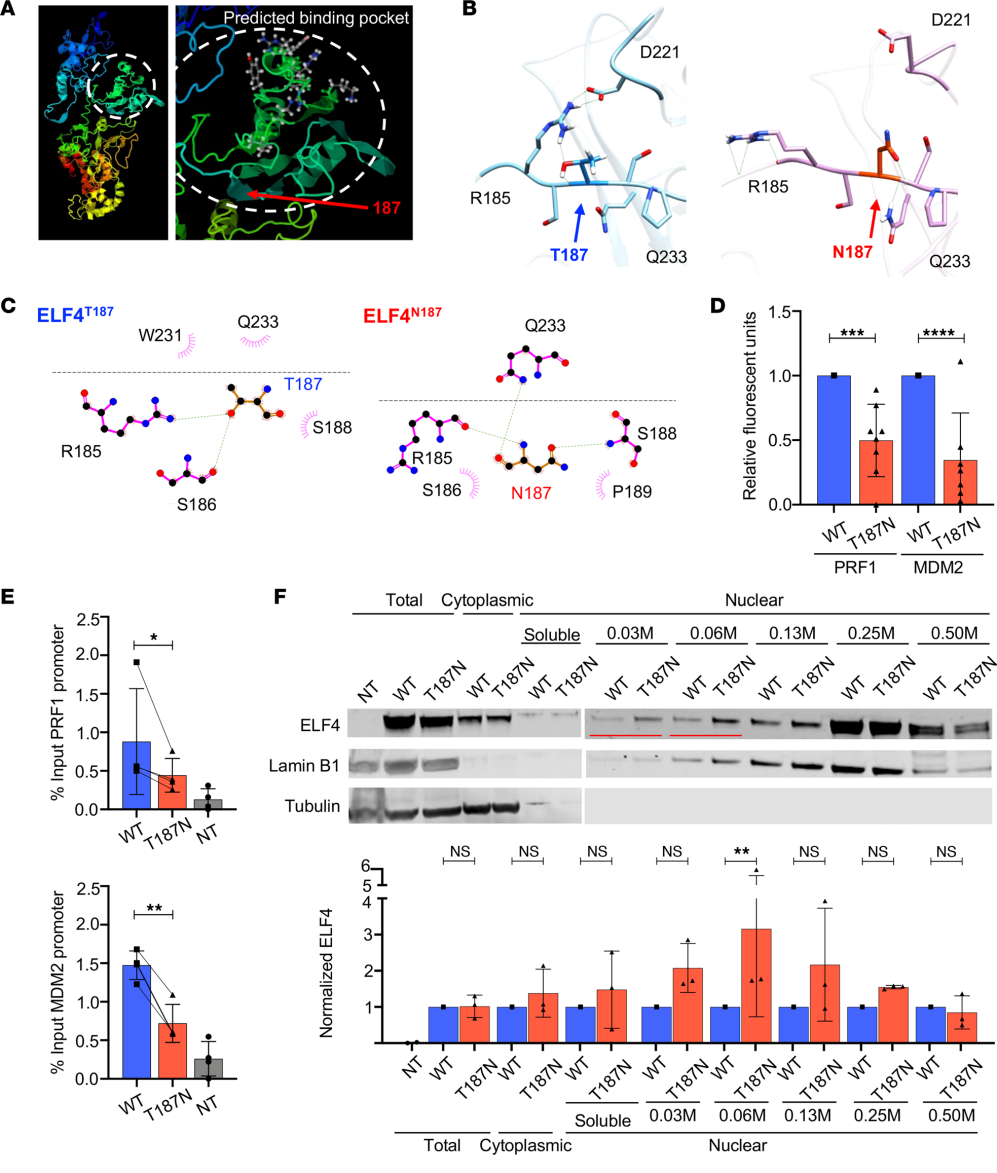
ELF4 functional changes induced by p.T187N. (**A**) I-TASSER prediction of ELF4 structure showing the backbone with a cluster predicted to form a binding pocket encircled and DNA binding residues with red arrow pointing at aa 187. (**B**) UCSF Chimera software analysis from predicted models of hydrogen bonds (green lines) with listed aa residue interactions of ELF4^T187^ (blue, left) and ELF4^N187^ (pink, right). (**C**) LIGPLOT software analysis from predicted models of Van der Waals interactions (pink) and hydrogen bonds, showing predicted interactions with aa within the same chain or proximal (across the black line) based on protein folding for ELF4^T187^ and ELF4^N187^. (**D**) Relative fluorescence units of luciferase promoter reporter (PRF1 and MDM2) for ELF4 T187N overexpression normalized to the WT control. (**E**) Independent repeats of ChIP quantitative PCR for overexpressed WT and T187N ELF4 bound to promoters (NT, nontransfected). (**F**) Salt-titrated extraction assay of chromatin-bound ELF4. Western blot run on a gel including the total cell lysate and the cytoplasmic and nuclear soluble fractions and a second gel including the chromatin-bound salt extraction. Showing the proteins detached with the increasing concentrations of salt from the chromatin-bound fraction after sequential extraction of the cytoplasmic, nuclear soluble, and chromatin-bound fractions (top, representative blot) with red lines indicating the significant difference in extraction between the WT and T187N ELF4 at lower salt concentrations. ELF4 expression normalized to loading control and subsequently to the wild-type control (bottom) across 3 independent experiments. The data represent mean ± SEM of ≥3 independent experiments. Western blot is representative of experiment repeated at least 3 times; *P < 0.05, **P < 0.01, ***P < 0.001, 2-tailed paired Student’s *t* test and Wilcoxon matched pairs signed-rank test.

Given that ELF4 T187N is expressed and localized normally, we next asked if its DNA binding function was altered. Since the crystal structure of ELF4 has not been previously resolved and our missense variant introduces a substantive physiochemical alteration, we used the I-TASSER software to predict the variant’s impact on the protein structure (32). Interestingly, a binding pocket and DNA binding residues were predicted to localize near T187 (Figure 7A). Residues predicted to be involved in the structure of the binding pocket included 185 and 186, which are predicted to interact with residues 219, 226, 231, 232, 288, 289, and 290 within the ETS domain, in which the DNA binding amino acids are located (Supplemental Table 4). The T187N variant increased the predicted normalized B factor score, indicating increased regional instability (Supplemental Figure 6). To better visualize the potential impact, we analyzed predicted interactions of the T187 amino acid and any changes predicted for the N187 using UCSF Chimera and LIGPLOT software (33, 34). The T187 amino acid hydroxyl group was predicted to form 2 hydrogen bonds: one with the functional group of R185, which stabilizes the spatial location of this amino acid, resulting in the formation of 2 hydrogen bonds with D221 (Figure 7B), and the second to the backbone of S186 (Figure 7C). The variant N187 amide functional group was predicted to create a hydrogen bond with the backbone of S188, allowing the backbone of N187 to form 2 novel hydrogen bonds, one to the functional group of Q233 and the other to the backbone of R185 (Figure 7C). This effect, in turn, predicts that R185 would form other hydrogen bonds disrupting the previous binding to D221 (Figure 7B). Thus, the T187N variant was predicted to change the interactions of amino acids residing within an ELF4 structure necessary for DNA binding, causing binding instability, which could impair ELF4 function.

To test the implications of these predictions, we overexpressed ELF4 WT and T187N in HEK293T. ELF4 transcriptionally regulates expression of genes by directly binding to their respective promoters, including *MDM2* and *PRF1*, and of type I IFNs genes by recruiting to the STING−MAVS−TBK1 complex to induce *IFNA* and *IFNB* expression (15, 35). Thus, we evaluated ELF4 WT and T187N transactivation function by cotransfecting it with a luciferase reporter construct using known promoter targets perforin (*PRF1*) and mouse double minute 2 homolog (*MDM2*), an E3 ubiquitin ligase that functions as a p53 inhibitor. With similar overexpression levels of WT and T187N ELF4, the transactivation of both perforin and MDM2 promoters was significantly decreased by T187N (Figure 7D), demonstrating decreased transactivation of known ELF4-regulated promoters by the variant.

We next characterized the direct interaction of ELF4 and the impact of the T187N variant on its function using chromatin immunoprecipitation (ChIP) of the PRF1 and MDM2 promoters. We found that the ELF4 T187N construct significantly decreased binding to both promoters (Figure 7E). To further understand how the variant resulted in decreased DNA binding, we evaluated the impact of the ELF4 p.T187N variant strength on its binding to chromatin by performing a salt extraction assay. We overexpressed ELF4 WT or T187N constructs in HEK293T cells and fractionated cytoplasmic, nuclear soluble, and chromatin-bound fractions, then extracted proteins from the chromatin according to their binding strength by adding buffer with incremental NaCl concentrations (36). A significantly increased amount of the ELF4 T187N protein was extracted at lower NaCl concentrations (0.06 M) compared with WT (Figure 7F). Amounts of unbound ELF4 protein were normalized as the sample was saturated to extract all proteins (Figure 7F). Therefore, at a lower concentration of NaCl, there is decreased ELF4 binding in the presence of the T187N variant, suggesting its altered structural properties impair its function. Thus, the T187N variant is hypomorphic, defined by decreased ability to promote transactivation of gene targets important for NK cell function and development, likely causing the proband’s NKD.

## Discussion

Using complementary biochemical and functional assays from overexpression models, a mouse model, and primary human samples, we demonstrated that the ELF4 p.T187N variant is deleterious, resulting in an NKD phenotype. Following the guidelines (37) of Casanova et al., we have demonstrated that this variant is monogenic, is deleterious to the gene product, and has a causal relationship established via relevant models. As we modeled the pathogenicity of the ELF4 p.T187N variant, we gained insight into the complex transcriptional pathways and mechanisms by which ELF4 may contribute to the regulation of NK cells. We demonstrated that the ELF4 p.T187N variant is hypomorphic and of specific relevance to NK cells, and while it is beyond the scope of this project to elucidate all its roles, it causes a decrease in NK cell frequency, maturity, and perforin expression, consistent with an NKD.

We demonstrated that the variant, both in our mouse model and in the proband, results in decreased frequency of NK cells with decreased maturation, similar to the phenotype observed in *Elf4^–/–^* mice (15). The reduced absolute number of NK cells generated in vitro from proband CD34^+^ hematopoietic precursors suggests that hypomorphic ELF4 function contributes directly to the decreased frequency of NK cells found in the proband independently of any somatic, infectious, or inflammatory influences that might have been present in vivo. While it is possible ELF4 regulates the proliferation and differentiation of early NK cell development stages (23), we did not find differences in the generation of stages 1–3; however, the decreased maturation in the proband and the generation of terminally mature stage 5 NK cells suggests ELF4’s role is in terminal maturation. Similarly, *Elf4^–/–^* mouse NK cells and those expressing the ELF4^T187N^ variant demonstrated increased immature CD27^+^CD11b^–^ and a decrease in terminally mature CD27^–^CD11b^+^ and mature CD27^+^CD11b^+^ subsets. Thus, ELF4 is necessary for the normal development and maturation of NK cells (15), and the hypomorphic variant causes aberration.

Despite some plasticity (8) between stage 4 CD56^bright^ and stage 5 CD56^dim^ NK cells, their progenitor-progeny relationship has been questioned (38, 39). Since ELF4 levels correlate to activity, the substantial increase in ELF4 expression between CD56^bright^ and CD56^dim^ NK cells and the accumulation of CD56^bright^ cells in the proband suggests ELF4 functions in NK cell development at the linear transition from stage 4 to 5. Even though experimental evidence supports this progenitor-progeny relationship, it does not mean all CD56^bright^ cells are destined to become CD56^dim^ NK cells (40). Thus, distinct ELF4 expression in stages 4a and 4b could dictate maintenance of CD56^bright^ instead of maturation into CD56^dim^ NK cells. While it was beyond the scope of this study to fully dissect the mechanism underlying disrupted NK cell maturation, we hypothesize that the known role of ELF4 in the regulation of cell cycle and p53/MDM2 pathway (25, 31) may contribute. A similar phenotype of decreased NK cell terminal maturation is found in patients with MCM4 and GINS1 variants, which are known to impair cell cycle progression. That said, despite decreased transactivation of the MDM2 promoter by the ELF4 p.T187N variant, we did not observe a change in cell cycle or cell growth in the cell models.

We demonstrated that ELF4 is necessary for perforin transcription and optimal NK cell cytolytic function using multiple models. In the human NK cell lines, NK92 and YTS, disrupting ELF4 expression reduced nascent and processed perforin by 24% and 38% and by 10% and 28%, respectively, without altering granzyme B expression. Thus, ELF4 directly affects perforin independently of maturation, and the reduced perforin-positive cells in the proband and the ELF4^T187N^ murine NK cells reflect the hypomorphic variant disrupting perforin expression. This is further supported by decreased perforin in the proband’s T cells, which did not impact T cell cytolytic function, unlike that observed in the proband’s and ELF4^T187N^ murine NK cells. While our results demonstrate human ex vivo and NK cell line impact of ELF4 p.T187N, it is crucial to recognize that mice under SPF conditions have neonate-like immune systems, and comparisons between feral, pet store, and laboratory mice have shown impact upon cytolytic lymphocytes (41). Thus, further modeling using exposure to commensal and infectious pathogens will help us fully understand the extent to which the ELF4 p.T187N variant affects NK cell function and the immune response. Additionally, our focus on perforin and granzyme B does not consider other cytolytic mechanisms, which warrants further evaluation and, ideally, broad transcriptional profiling.

NKD occur in the classical sense when an individual has a gene defect that interferes with NK cell development, maturation, or survival, most of which affect NK cell transcription or cell cycle (1). As a result, cNKD lead to deficient NK cell function. We evaluated ELF4 as a potentially novel gene candidate for NKD, and it joins 2 other transcription factor genes, *IRF8* and *GATA2*, that, when aberrant, interrupt NK cell maturation (1). Notably, only particular variants cause NKD, owing to their specific impact. In IRF8 deficiency, at present, only biallelic variants have been described to cause NKD, and only those individuals with IRF domain variants seem to have a predominant NK cell phenotype (43). We identified ELF4 p.T187N as resulting in NK cell–focused dysregulation, with impaired ELF4 function resulting from decreased binding. Variants in different regions likely yield phenotypes in which NKD is not preferential and may affect other lymphocyte subsets, as suggested (44).

Additional mild abnormalities were noted in our cohort, including some hypogammaglobinemia, impaired vaccine responses, and B cell lymphopenia. IgG therapy given to the proband did not appear to affect the recurrence of viral infections. Further investigation is needed to identify the association between ELF4 T187N and B cell and immunoglobulin irregularities in our cohort. Previously, a family was described with X-linked hypogammaglobulinemia (XLA) having *ELF4* c.1487T>C p.S369P (45). Carrier females were mildly affected, and hemizygous males were more severely impacted. While cosegregation of this variant with XLA was clear, evidence for impact upon transcription or a mechanism for pathogenesis in B cells was not provided (45). Importantly, *Elf4^–/–^* mice have normal B cell proliferation (15); thus, further studies are needed regarding the pathogenesis of ELF4 p.S369P. Recently other *ELF4* variants that abrogated expression and led to immune dysregulatory phenotypes were reported (44). A spectrum of diseases and phenotypes associated with different variants of a single gene is not uncommon in IEI, with *ELF4* seeming to be no exception.

The description of additional patients often corroborates the initial report of single patients (37). While initially studying a single proband, we subsequently identified a second family with the same variant and thus felt compelled to extend our study to include this case. Individual I-1 in this additional family seemed to have an IEI phenotype with notable cancer susceptibility. Little information is available regarding the status of his NK cells since limited studies were performed before his death; however, his medical history of lymphoproliferative disease and lymphoid malignancies could suggest an NK cell abnormality. Given the role of NK cells in immune surveillance and activity against cancer, patients with NKD may be more susceptible to the development of malignancies (46).

The contribution of ELF4 to cell fate will likely depend on the environmental and cellular context, given its critical roles in cell cycle regulation and cellular homeostasis (30). Exposure to specific pathogens could trigger different phenotypes in patients carrying *ELF4* variants, even the same variant, and pathogen-free experimental environments can be mechanistically limiting. Further work is needed to understand the relationship of *ELF4* variants with malignancy and immune dysregulation and to assess the effect of commensal and infectious pathogens. Moreover, further investigation is necessary to understand the impact of the variant in the heterozygous female individuals having either a decrease in NK cell frequency or decreased cytolytic response to IL-2 stimulation (reflecting proband phenotype). Due to a lack of access to additional family members, we could not address the penetrance of the variant. Nonrandom X inactivation analysis would be important to pursue in subsequent studies of *ELF4* to understand the effect on heterozygous carriers and strengthen the correlation between the variant and disease phenotype.

Our study demonstrated that ELF4 p.T187N causes an NKD affecting both NK cell development and function. We determined that the p.T187N variant is rare, monogenic, damaging, located in a conserved residue, and disruptive of the normal function of ELF4. Through a variety of in vitro human cell assays and a murine bone marrow chimera model, we showed the p.T187N ELF4 variant in NK cells impaired perforin expression, impeding function, and disturbed normal NK cell development and maturation. Unlike other classical NKD genes whose functional abnormalities are due to NK cell developmental defects, the functional impairments in the NK cells expressing our variant are due to an independent functional effect. Thus, the *ELF4* variant c.560C>A p.T187N is a hypomorphic variant that damages NK cells by disrupting normal NK cell development and through a potential block in maturation and further exacerbates the impact on NK cells by additionally affecting function.

## Methods

All catalog numbers, antibody clones, cell line information, mouse information, and additional details can be found in the Supplemental Methods.

### Whole-exome sequencing and analysis.

After enrollment in the Baylor-Hopkins Center for Mendelian Genomics IRB protocol H-29697 at Baylor College of Medicine and the NK Cell Evaluation and Research Program at Baylor College of Medicine (BCM) and Columbia University Irving Medical Center (CUIMC), whole-exome sequencing (WES) was performed on the proband’s genomic DNA, extracted from EDTA-preserved blood. WES was performed as described (47) on the proband’s genomic DNA, extracted from EDTA-preserved blood. Bioinformatic filters were used (48, 49) for the final selection of candidate gene variants as described in the Supplemental Methods. We have deposited the individual variant to ClinVar (accession number SCV002599127).

### Sanger sequencing.

Variants were confirmed by Sanger sequencing on genomic DNA extracted from blood using primers designed with Primer-3. PCR amplicons were sequenced by the Baylor DNA Sequencing Core Facility and visualized with 4-Peaks software.

### PBMC isolation, NK cell enrichment, and T cell expansion.

All human blood samples were obtained under protocols approved by the IRB for the Protection of Human Subjects of BCM and CUIMC. PBMCs were isolated and NK cells and CD34^+^ cells were enriched as described (3). T cells were expanded from PBMCs by culturing them in R10 media with phytohaemagglutinin and IL-2 for the first week, followed by once-weekly stimulations.

### Tonsil processing.

Tissue from routine tonsillectomies, obtained under IRB approval from CUIMC, was used to isolate cells by density centrifugation after mechanical separation with a 40 μm filter (Thermo Fisher Scientific).

### In vitro NK cell differentiation.

In vitro NK cell maturation assay was performed as described (3, 49, 50) using CD34^+^ precursors enriched from the peripheral blood via human hematopoietic progenitor cell enrichment cocktail (Stemcell Technologies) and further purified by FACS. Following purification, cells were incubated on mitotically inactivated EL08.1D2 stromal cells in media containing cytokines as described (3) and phenotyped by FACS.

### Mice.

*Elf4^–/–^* mice were backcrossed for more than 12 generations to the C57BL/6J background. All mice were bred and maintained under SPF conditions. All experiments were performed with approval of BCM’s Institutional Animal Care and Usage Committee.

### Mouse model.

Generation of bone marrow chimera was as described (51) in C57BL/6J mice. Retroviral constructs were generated by Epoch Life Science custom cloning services. Bone marrow cells were collected, cultured, transduced with ELF4^WT^ and ELF4^T187N^ retrovirus, purified by cell sorting, and injected intravenously into lethally irradiated *Elf4^–/–^* or *Elf4^+/+^* recipients. Three months later, blood was collected to confirm NK reconstitution, and mice were euthanized to collect spleens 24 hours poststimulation. Tissue from mice was processed on ice, and cells were isolated by mechanical separation using a 40 μm strainer (Thermo Fisher Scientific). RBCs from the single-cell suspension pellet were lysed by deionized water, followed by quickly recovering isotonic conditions with 4× PBS. Additional details can be found in the Supplemental Methods.

### Cell lines.

Cell lines were maintained in complete media as indicated in Supplemental Methods. YTS, BLCL, K562, 721.221, Raji, P815, and YAC-1 cell lines were cultured in RPMI-1640 (Thermo Fisher Scientific), HEK293T cells in DMEM (Thermo Fisher Scientific), and NK92 cell lines in Myelocult (Stemcell Technologies) (52). BLCL were generated from patient PBMCs by adding EBV supernatant. Cell lines were confirmed mycoplasma negative before their use in experiments.

### Cell cycle.

Cell cycle was assessed using BrdU Flow Kit (BD Biosciences) per recommended protocol. Stages of the cell cycle were evaluated via imaging flow cytometry by plotting DAPI versus BrdU.

### Inducible KD cell line production.

Cultured YTS and NK92 cells were transduced by centrifugation at 1,290*g* for 90 minutes at 32°C with SMARTvector Lentiviral shRNAs from Dharmacon using TransDuxMax (Systems Biosciences). After 2 weeks, shRNA was induced using doxycycline 1 μg/μL for 48 hours, and cells were sorted based on their GFP expression to establish a stable cell line. Subsequently, cells were split into experimental samples treated with or without doxycycline (2 μg/μL) for at least 2 weeks.

### CMA treatment.

Doxycycline-inducible shRNA NK cell lines previously established and validated were used. Cells were subsequently treated for 30 minutes with 100 nM CMA, washed, and incubated for 48 hours before use to phenotype cell lines.

### Cytotoxicity assay.

NK cell and T cell cytotoxicity were evaluated by ^51^Cr release assay as described (53–56) using YAC-1, K562, Raji, 721.221, or P815 as target cells for 4 hours at 37°C. Cytotoxicity mediated by expanded T cells was measured using P815 target cells preincubated with anti-CD3 (56).

### LU calculation.

LU were calculated as previously described (55). LU are defined as the number of effector cells required to mediate lysis of a specific percentage of target cells expressed as the inverse normalized to cells. From cytotoxicity curves plotted into 1-phase association, nonlinear fit best-fit values were calculated. LU were calculated for NK cells sorted from murine spleen as the inverse of 1 million cells required to lyse 5% of target cells and for PBMCs and T cells as the inverse of 1 million cells required to lyse 10% of target cells using.01 million targets per well. The latter was normalized to the percentage of NK cells in the PBMC sample and subsequently the percentage of CD56^dim^ NK cells in the total NK cells as shown below.


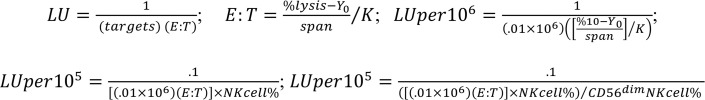


### Immunofluorescence.

For surface staining without stimulation, washed cells were incubated in 100 μL PBS 2% FBS buffer with antibodies for a total volume of 200 μL for 15–20 minutes. Cells were permeabilized using Cytofix/Cytoperm buffer to detect intracellular markers or using Foxp3/TF Staining Buffer Kit to detect intracellular markers and transcription factors. When staining for ELF4, antibody was incubated for 45–60 minutes, followed by secondary antibody along with a cocktail of conjugated intracellular antibodies for 20–30 minutes. At the end of staining, cells were fixed in 1× PBS 1% paraformaldehyde and stored at 4°C until acquisition.

Tonsil samples or samples from in vitro development assays were used to assess NK cell stages using an NK cell differentiation panel (3); freshly isolated samples from mice were used to assess NK cell phenotype; cell lines were collected from their respective conditions to assess ELF4 and cytolytic proteins; and expanded T cells were used to assess their phenotype for which the antibodies can be found in the Supplemental Methods.

Freshly isolated or cryopreserved PBMCs were used to assess NK phenotype using activation, adhesion, inhibitory, development, inhibitory, and cytokine validated panels as previously described (20).

### FACS and flow cytometry.

Cells were purified using flow cytometric cell sorting BD FACSAriaII and S3e cell sorters. From PBMC samples and apheresis products, NK cells were sorted by CD3^–^CD56^+^ and precursors by Lin^–^CD34^+^ fluorescence. NK cells from murine splenocytes were sorted by CD3^–^NK1.1^+^ and GFP^–^ and GFP^+^ fluorescence. Inducible KD NK cell lines were sorted based on total GFP^+^ fluorescence. All cells were cultured with antibiotics after sorting.

LSRFortessa and FACSCelesta were used to acquire single live cells for phenotypic analysis. Positive populations were identified as those with fluorescence greater than the isotype or fluorescence-minus-one controls. Results were subsequently analyzed with FlowJoX.

### Imaging flow cytometry.

Cell images were acquired on an AmnisMKII imaging flow cytometer with a 60× objective (57). Nuclear localization was calculated as (Intensity_Nuclear Mask_Ch ELF4/Intensity_Cellular Mask_Ch ELF4) × 100. To create figure graphs, samples were merged into a single analysis file. Additional details on the analysis can be found in the Supplemental Methods.

### Transfection.

Cells were transfected using FuGene per manufacturer’s protocol with 2 μg of DNA in a 6-well plate. HEK293T were used to express ELF4 constructs. ELF4 IRES GFP constructs were used for luciferase assay, and ELF4-myc–tagged constructs were used for ChIP; both were used for fractionation and salt extraction assay. For luciferase assays, ELF4 WT or ELF4 T187N variant constructs were cotransfected with LightSwitch Promoter Reporter Vectors and quantified per manufacturer’s instructions. Relative fluorescence units were calculated as target promoter minus background divided by control minus background, normalized to the WT sample.

### ChIP-qPCR.

HEK293T cells were transfected with myc-tagged ELF4 WT or ELF4 T187N plasmids, and cell pellets were cross-linked with 1% formaldehyde and quenched with glycine before lysing. Lysates were sonicated using CovarisE220 focused ultra-sonication (target of 500 bp) and efficiency confirmed by agarose gel electrophoresis. The sheared chromatin was diluted, incubated with binding control for preclearing, followed by anti–myc-tag nanobody bound to magnetic beads overnight, after which magnetic separation was performed. Separated samples were washed with cold buffers 1–3 and then TE. Proteins were then de-cross-linked from the beads using elution buffer overnight at 65°C with the addition of NaCl and RNase. This was followed by incubation at 45°C with EDTA and proteinase K, and DNA was then purified using a Zymo Research PCR purification kit. Quantitative real-time PCR was performed using Custom Plus TaqMan RNA Assays (Thermo Fisher Scientific) to quantify the binding of ELF4 to *PRF1* and *MDM2* promoters. Results were analyzed using the percentage of input method: 100 × 2^Adjusted^ ^input^^–^^Ct^ ^(IP)^.

### Cell fractionation and salt extraction.

HEK293T cells transfected with *ELF4* plasmids were used 48 hours posttransfection for cellular fractionation and sequential salt extraction as previously described (58). Total cell lysate and cytoplasmic and nuclear fractions were prepared using NP-40 and cell extraction buffer with NP-40 buffers. Salt extraction buffer consisting of serial dilutions of 0.5 M NaCl in nuclear extraction buffer was added starting at 0.03 M, and supernatants were collected and frozen for subsequent Western blot analysis.

### Western blot.

Cells were lysed with RIPA lysis buffer, prepared with LDS and denaturing agent, separated via PAGE, and transferred to nitrocellulose membranes. Membranes were probed for ELF4 and successively with tubulin (for total and cytoplasmic loading control) or Lamin B1 (for nuclear loading control). ELF4 protein expression was calculated by normalizing to loading control with subsequent normalization of ELF4 to determine expression of T187N relative to WT.

### Protein modeling and analysis.

The I-TASSER server (32) was used to generate predicted full-length 3D models from the ELF4^WT^ and ELF4^T187N^ protein sequences.

LIGPLOT (33) and UCSF Chimera (34) software were used for protein structure visualization and intermolecular measurements, as well as to illustrate putative hydrogen bond network rearrangement and conformational changes.

### Statistics.

Data are represented as means ± SD of ≥3 biological replicates. Statistical analysis was performed using GraphPad Prism 9.1.0. Statistical comparisons were performed using 2-tailed Student’s *t* tests with Bonferroni’s correction for multiple comparisons, paired Student’s *t* tests, 1-way ANOVA, and 1-way repeated measures ANOVA and Wilcoxon matched pairs signed-rank test where specified. A *P* value less than 0.05 was considered significant, and different value thresholds were noted.

### Study approval.

All studies were performed in accordance with the Declaration of Helsinki with the written and informed consent of all participants under the guidance of NIH, Children’s Hospital of Philadelphia, Seattle Children’s Hospital, BCM, and CUIMC, under the IRB protocols CHOP 2006-7-4885, BCM H-30487, and CUIMC AAAR7377 and AAAQ0156. Additionally, written informed consent was provided to include patient pictures. Animal studies were performed in accordance with the BCM and NIH Animal Care Committees.

## Author contributions

SAS, EMM, MIC, CSP, YL, JIRS, AER, SM, and JSO designed research studies, conducted experiments, and collected and analyzed data. BJL, EKM, and AF conducted experiments. IKC, RAG, JEP, and JRL performed genetic analysis. ERH was clinical coordinator for sample collection. ERH, AT, LRFS, and EJA collected clinical data. SAS, EMM, MIC, CSP, LRFS, JIRS, EJA, TGW, HDL, and JSO wrote or revised the manuscript.

## Supplementary Material

Supplemental data

## Figures and Tables

**Table 1:**
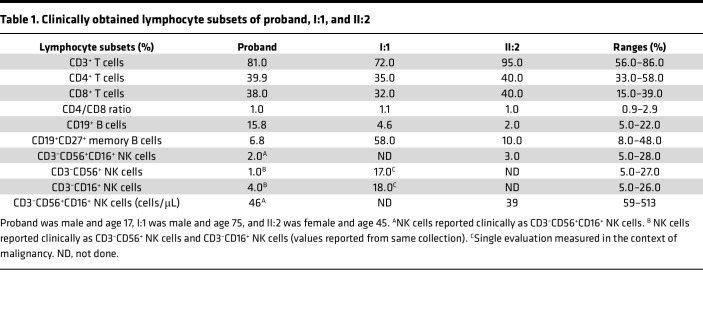
Clinically obtained lymphocyte subsets of proband, I:1, and II:2

## Version 1 12/08/2022

Electronic publication
