# Supplementary material for: An ELF4 hypomorphic variant results in NK cell deficiency
Source: JCI Insight. 2022 Dec 8;7(23):e155481. doi: 10.1172/jci.insight.155481 (PMC9746917; doi:10.1172/jci.insight.155481)
Supplement: Supplemental data [file jciinsight-7-155481-s188.pdf]

Supplemental Figures

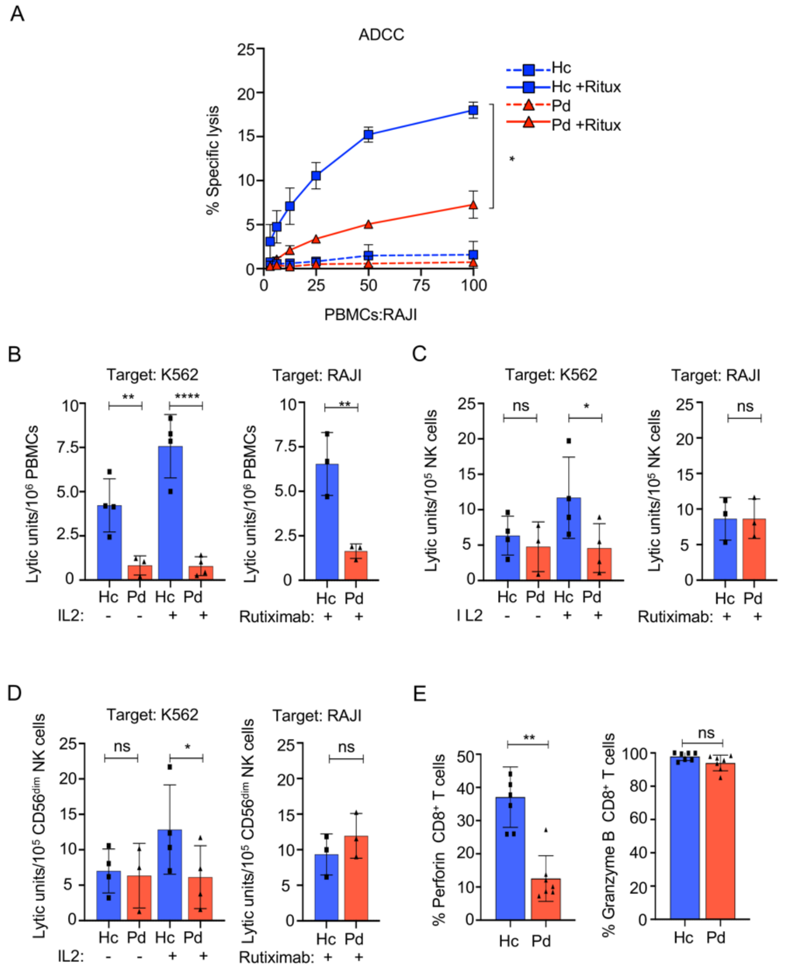

Supplemental Figure 1**. Cytotoxicity lytic units' normalization. (A)** ^{51}Cr-release assay using PBMCs against Raji target cells opsonized with rituximab testing NK cell ADCC. **(B)** Lytic units calculated from the NK cytotoxicity assays with and without IL2 stimulation and ADCC. (**C, D**) LU from the NK cell cytotoxicity and ADCC were normalized to the percent of NK cells per sample and subsequently normalized to the percent of CD56^{dim} NK cells, respectively. (**E**) Frequency of perforin and granzyme B positive CD8^{+} T cells from different repeats and time points after T cell stimulation. Data represent ≥3 biological replicates; not significant (ns)≥.05, **P*<.05; 2-tailed Student *t*-test.

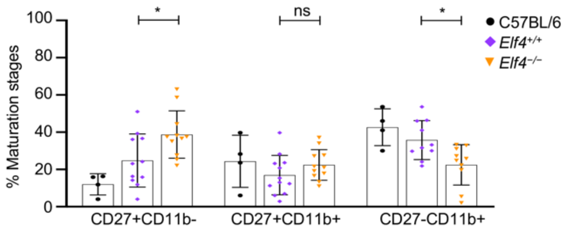

Supplemental Figure 2. **Reduced NK cell maturation in *Elf4* knockout mice.** NK cell maturation subsets from spleen samples. NK cells from control (black), *Elf4*$^{+/+}$ (purple) and *Elf4*$^{-/-}$ (orange) mice. Data represent 3 independent experiments; each symbol represents an individual mouse with an *N* of ≥4; not significant (ns)≥.05, **P*<.05; 2-tailed Student *t*-test.

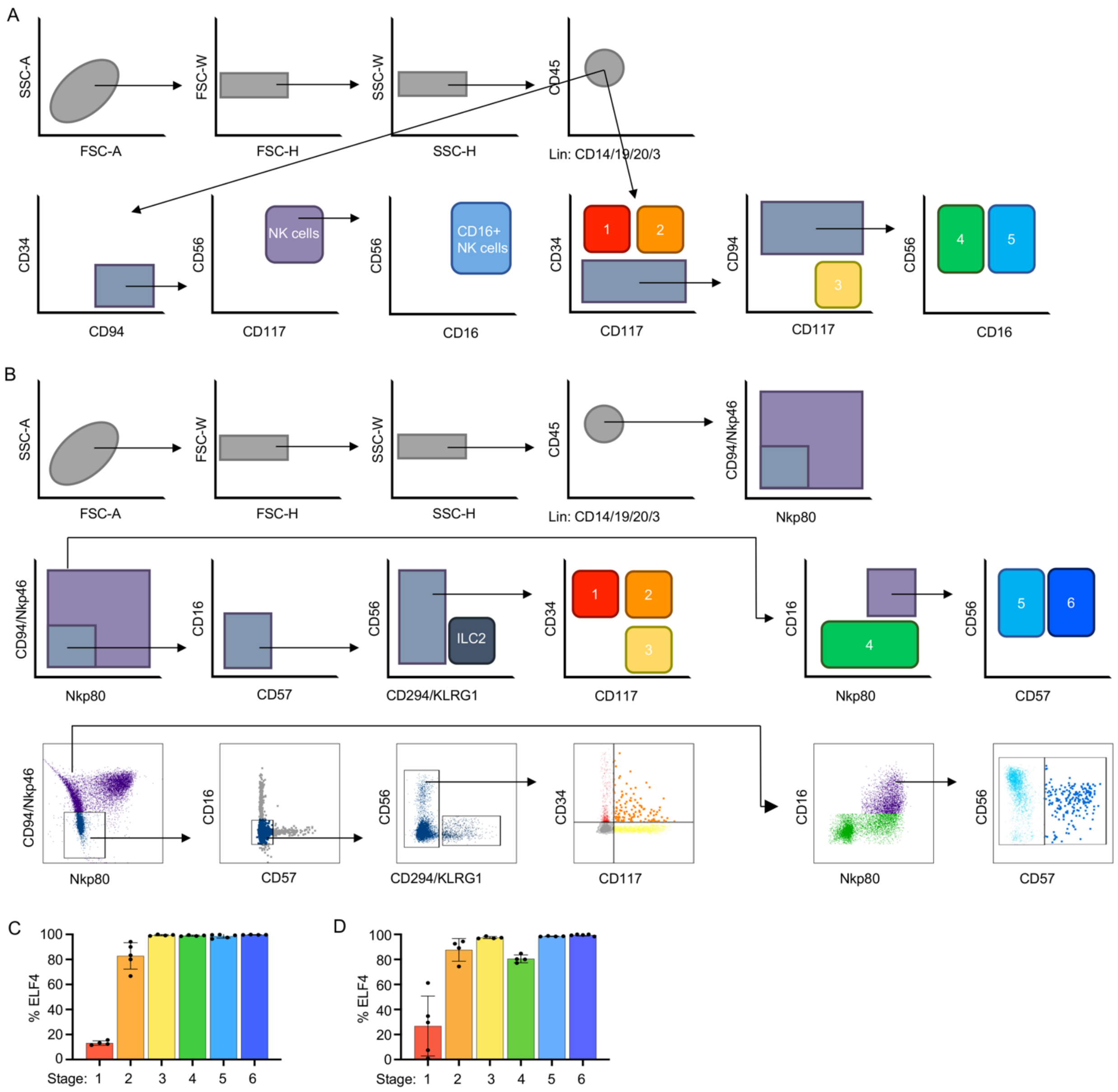

Supplemental Figure 3. **Gating human NK cell intermediates and ELF4 expression in NK cell stages.** (**A**) Analysis of *in vitro* NK cell differentiation assay to identify the frequency of NK cells produced, and mature population to identify the frequency of developmental stages generated at the end of the assay. (**B**) Gating strategy and representative plots to identify NK precursors from human samples. (**C**, **D**) ELF4 positive frequency of NK precursors (**C**) PBMCs

(**D**) Tonsils. Data represent $n=5$; $^*P<.05$, not significant (ns) $\geq.05$; 2-tailed Student t-test with multiple comparisons and ANOVA.

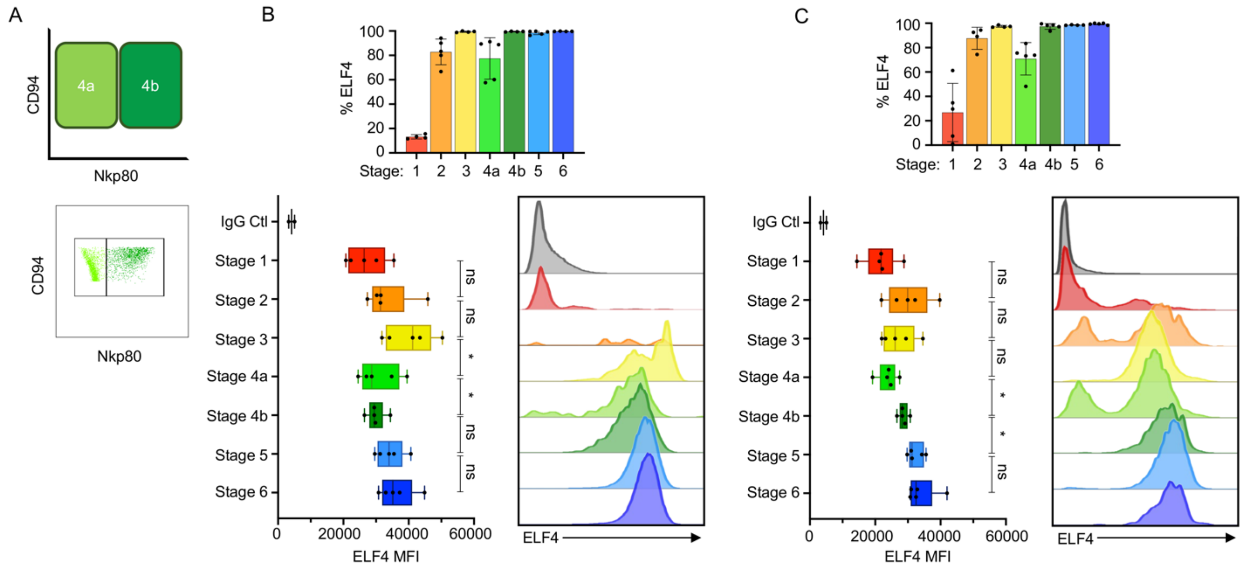

Supplemental Figure 4. **Gating human stage 4a and 4b NK cell intermediates and ELF4 expression in them.** (**A**) NKp80 distinguishes Stage 4a from 4b (**B**, **C**) ELF4 expression in NK precursors, including stages 4a and 4b from human samples (**B**) PBMCs (**C**) Tonsils. Data represent *n*=5; **P*<.05, not significant (ns) ≥.05; 2-tailed Student *t*-test with multiple comparisons and ANOVA.

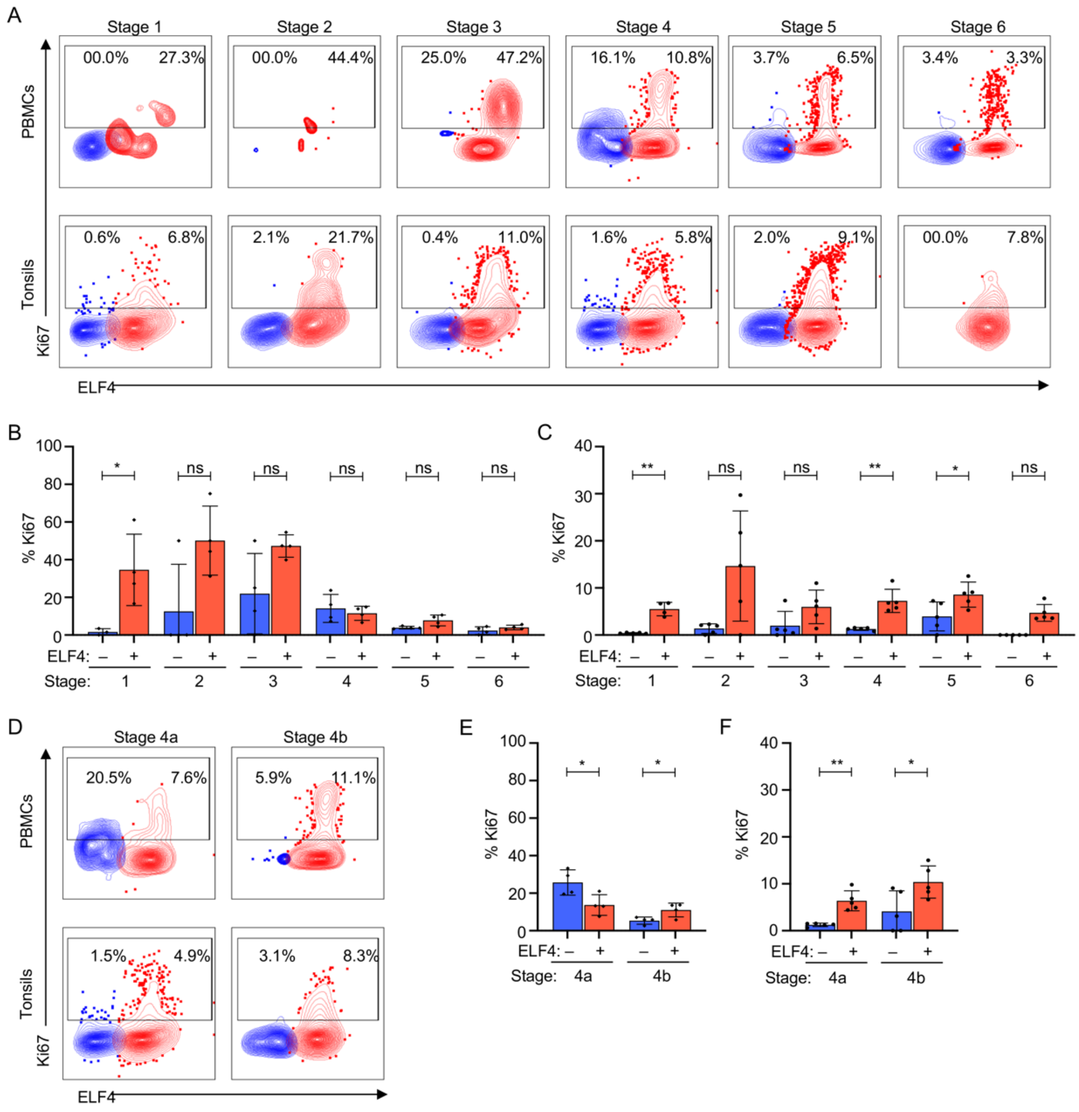

Supplemental Figure 5. **Expression of Ki67 and ELF4 MFI in ELF4 positive human NK cells.** (**A**) Representative plot of Ki67 expression in NK cell intermediate stages from PBMC and Tonsil samples. (**B**, **C**) Frequency of $Ki67^{+}$ cells from $ELF4^{+}$ (red) and $ELF4^{-}$ (blue) NK cell precursors in PBMCs (**B**) and Tonsils (**C**). (**D**) Representative plot of stages 4a and 4b. (**E**, **F**) Ki67 frequency in $ELF4^{+}$ and $ELF4^{-}$ stages 4a and 4b in PBMCs (**E**) and Tonsils (**F**). Data represent *n*=5; **P*<.05, not significant (ns) ≥.05; 2-tailed Student *t*-test with multiple comparisons and ANOVA.

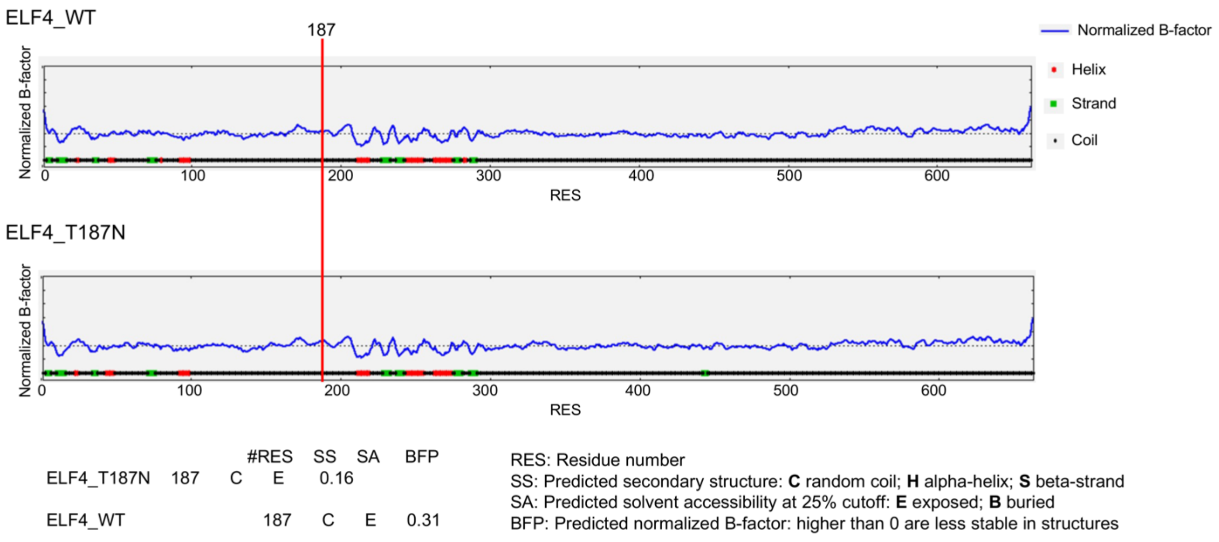

Supplemental Figure 6. **Predicted decreased ELF4 variant stability.** I-tasser predicted normalized B-factor, indicating the approximate location of aa 187 and its corresponding reported B-factor profile.

Supplemental Tables

	Summary of Clinical History		Shared Findings	Research Findings
	Immunologically relevant	Additional Medical History		
Proband	Male child with a history of lower respiratory tract infections and multiple ear infections requiring tympanostomy tube placement, tonsillectomy, and adenoidectomy before age 2, at age 4, developed severe primary EBV infection. By age 7, he was diagnosed with Von Willebrand's disease and was hospitalized for pneumonia. Diagnosed with mild hypogammaglobulinemia due to poor vaccine-specific IgG responses, started treatment with immunoglobulin replacement. Developed zoster with reported lesional viral detection (although the actual records were no longer maintained in hospital archives) with recurrent outbreaks treated with high dose valacyclovir. T and B lymphocyte subsets were within normal ranges with normal subset distribution.	Patient with history of autism and nephrolithiasis, secondary to the aforementioned conditions, a gastrostomy tube was placed to allow for supplemental and overnight hydration.	Mild hypogammaglobulinemia (IgG 584 mg/dL, IgA 85 mg/dL, IgM 54 mg/dL, IgE 12 kU/L)<br><br>Clinically NK cell frequency and absolute Number were reduced.	Decreased NK cell frequency<br>Increased Immature NK cells<br>Decreased NK cell cytolytic function
Proband's Mother	Mother reports to be generally well but had marginal low IgG subclass levels and recurrent sinus infections.	No additional history provided.	Marginal low IgG subclass levels<br><br>NK cells' clinical information was not provided.	Normal NK cell frequency<br>Normal Immature NK cell frequency<br>Normal cytolytic function with a decrease in cytolytic function when stimulated with IL2
II:2	Female individual with hypogammaglobulinemia (IgG 15 mg/dL, IgA 2 mg/dL, and undetectable IgM and IgE) with absent titers against diphtheria was diagnosed with common variable immunodeficiency (CVID) at age 45 with a history of recurrent sinusitis since age 9 and lymphoproliferative phenotype including Granulomatous Lymphocytic Interstitial Lung Disease (GLILD), splenomegaly, and reactive peripheral lymphadenopathy.	No additional history provided.	Hypogammaglobulinemia (IgG 15 mg/dL, IgA 2 mg/dL, and undetectable IgM and IgE)<br><br>Clinical evaluation revealed mild lymphopenia with decreased NK frequency and absolute number	Decreased NK cell frequency<br>Normal Immature NK cell frequency<br>Normal cytolytic function with a decrease in cytolytic function when stimulated with IL2
I:1	Male individual with a medical history of recurrent oral ulcers and other infections, including frequent purulent otitis media with ruptured tympanic membranes, repeated bacterial sinusitis and pneumonia, and one episode of bacteremia. Immunophenotyping revealed mild B cell lymphopenia elevated memory B cells, decreased IgG and IgA class-switched memory B cells, a lack of immature/transitional B cells, and expanded CD38neg CD21low anergic/activated B cells. IgG, IgM, and IgA levels were normal at 921, 190, and 126 mg/dL, respectively. Specific antibody responses showed protective titers to tetanus (>2.24), Diphtheria (0.37), and pneumococcus titers (17/23 serotypes). He had a diagnosis of the following recurrent malignancies.<br>a) Lymphomatoid papulosis, a rare form of indolent cutaneous T-cell lymphoma characterized by crops of recurrent self-healing papules, which were treated with topical steroids to decrease the time of papule resolution.<br>b) Two distinct cutaneous CD30+ anaplastic large T cell alk-negative non-Hodgkin lymphomas. These were diagnosed at age 62 with initial treatment, including CHOP (cyclophosphamide, doxorubicin, vincristine, and prednisone) and radiation therapy leading to a remission that lasted until age 74. The remission was initially treated with 6 cycles of bendamustine with an incomplete response, followed by treatment with gemcitabine, including denosumab and pegfilgrastim for hypercalcemia and neutropenia, respectively.<br>c) Recurrent angioimmunoblastic T cell lymphoma (AITL)<br>Diagnosed concurrently and treated with the aforementioned non-Hodgkin lymphomas. The patient ultimately died from complications from the AITL with encephalopathy, hypoxemia, and hyperuricemia.	No additional history provided.	Mild B cell lymphopenia and B cell immunophenotyping revealed elevated memory B cells, decreased IgG and IgA class-switched memory B cells<br><br>Clinical evaluation revealed mild leukopenia with standard NK cells.	No research evaluation was possible

Supplemental Table 1. **Medical History**. Summary table of proband, proband's mother, I:1, and II:2 medical history and research findings.

	Proband	I:1	II:2	Normal Range
Sex	M	M	F	
Age	15.00	75	45	
**Absolute Lymphocyte Subsets**	(cells/mcL)			
CD3+ T cells	1230	1045	847	700-2100
CD3+CD4+ T cells	575	508	568	300-1400
CD3+CD8+ T cells	647	465	268	200-900
CD19+ cells B cells	105	73	79	110-500
CD16/CD56 NK cells	46	ND	39	59-513
**Lymphocyte Subset**	(%)			
CD4+ T cells (% of total lymph)	41.6	35	40	28-57
CD4+CD45RA+CD27+CCR7+ T cells (% of CD4+)	25	ND	3	3-33
CD8+ T cells (% of total lymph)	46.8	32	40	42-73
CD48CD45RA+CD27negCCR7neg TEMRA cells (% of CD8+)	ND	ND	8	0-10
CD4:CD8 ratio	0.89	1.09	1	1.0-3.6
CD16+CD56+ NK cells	2	ND	3	4-28
CD19 B cells (% of lymph)	7.7	4.60	11	6-19
Memory B cells (of CD19)	43.6	58.00	10	8-33
Naïve B cells (of CD19)	ND	42	90	48-95
Mature Memory (of CD19)	ND	53	10	8-48
Functional Mature Naïve B cells of CD19	ND	44	85	48-95
Immature/Transitional B cells of CD19	2.5	0.2	4.8	0.6-9
IgM IgD class-switched B cells of CD19+CD27+	6.0	18	4	0-24
IgM IgD class-switched B cells of CD19+	5.7	10.5	0.3	6-30
Anergic Activated B cells (of CD19)	ND	12.6	16.5	0-10
CD19+CD27+ IgG c-s Memory	ND	3.0	1.5	10-80
CD19+CD27+ IgA c-s Memory	ND	4.1	0.1	5-40
IgG:IgA ratio	ND	0.7	12.0	0.5-3.5
Plasmablasts	0.1	1.2	0.0	0-4

ND: not done

Supplemental Table 2. **Expanded clinical results**. Additional lymphocytes subset clinical results from proband, I:1, II:2.

	Raw_gene_name	Zygosity	CHROM	POS	REF	ALT	CMG_freq	esp5400_freq	1000_GA_freq	ExAC_ALL	CADD_phred	pLI	GDI_Phred
Homozygous missense	*ELF4*	Hom	X	129205367	G	T	0.000235923	0.000114142	0	0.00007013	17.7	0.611464084	47.88
Heterozygous deleterious	*ATAD2*	Het	8	124382158	TTCATCATCA	T	0.002123309	0	0	0.003	.	0.999997953	150.87
	HDAC1	Het	1	32797830	AGAG	A	0.001336898	0	0	0.0006	.	0.93791699	496.45
	KIAA0930	Het	22	45608215	G	A	4.33E-03	0.00278966	0.0046	3.80E-03	23.3	0.815289887	22.56
Heterozygous missense	*DGKZ*	Het	11	46397108	C	T	7.86411E-05	0	0	6.04E-05	24.3	0.998142634	3430.85
	EIF5B	Het	2	99995845	A	G	0.000157282	0	0	0.00005883	23.8	0.895387513	107.38
	ERP44	Het	9	102778676	A	C	3.70E-03	0.002881577	0.0014	3.30E-03	22.2	0.956751213	28.08
	EXOC3L2	Het	19	45731515	G	A	7.86411E-05	0	0.00E+00	0.00007505	21.9	0.925564032	776.33
	GRIK5	Het	19	42525579	C	T	0.001966027	0.001952036	0.0009	1.90E-03	27.1	0.917779688	136.49
	LRP1B	Het	2	141032113	C	G	2.36E-04	0.00E+00	0.00E+00	2.48E-05	27.8	0.99999919	3259.73
	NR4A1	Het	12	52450380	G	A	7.86E-05	9.29541E-05	0.0009	5.78E-05	34	0.959497621	439.58
	NTRK3	Het	15	88679172	C	T	7.86411E-05	0	0	0.000008241	23.4	0.978536238	54.71
	OSBPL6	Het	2	179209026	C	T	7.86411E-05	0	0	0.0002	17.61	0.999012304	690.82
	REV3L	Het	6	111696203	T	C	0.000865052	0.000464943	0.0009	0.0009	24.4	1	1709.51
	RNF220	Het	1	45110690	C	T	0.000786411	0.000929541	0	8.00E-04	25	0.999277879	77.57
	RPTOR	Het	17	78854273	C	T	0.000235923	1.86E-04	0	6.63E-05	32	0.999999983	64.75
	SBNO1	Het	12	123832641	C	T	0.000235923	9.29541E-05	0	0.00007475	26.1	0.999999984	1461.41
	SENP6	Het	6	76388558	G	A	0.000471846	0.000104471	0	0.0003	33	0.999876822	1761.81
	SMG7	Het	1	183514098	C	A	0.004482542	0.003904071	0.0023	0.004	28.4	0.999981493	2354.25
	TSC22D2	Het	3	150128839	G	T	0.002280591	0.001208403	0	0.0014	23.3	0.831881107	659.09
	UBAP2	Het	9	33923867	G	A	0.001258257	0.001208403	0.00E+00	0.0011	24.7	0.98950935	2836.17
	USP47	Het	11	11964069	A	G	0.00070777	0.001554726	0.0005	1.00E-03	19.43	0.999999875	110.16
Autosomal dominant heterozygous deleterious	*ITPR1*	Het	3	4726749	C	T	1.57E-04	0.000306937	0	8.55E-05	.	1	228.28
	LRP5	Het	11	68193442	G	T	7.86411E-05	0	0.00E+00	0.00002514	.	0.99835557	2445.61
Autosomal dominant heterozygous missense	*DNAJC13*	Het	3	132247160	T	G	2.75E-03	0.002602714	0.0005	2.30E-03	28.4	1	851.58
	GLI2	Het	2	121708913	G	A	7.86411E-05	0	0	0.000008291	23.2	0.998939596	484.45
	MYH11	Het	16	15834012	C	A	7.08E-04	0.000557724	0	4.00E-04	22.2	0.998956488	2033.85
	TBX1	Het	22	19751796	G	A	7.86E-05	0	0	0.00001668	32	9.83E-01	1845.84
Compound heterozygous deleterious	*TNRC6A*	Het	16	24788405	GCAGCAGCAGCCA	G	7.86411E-05	0.00E+00	0	3.00E-04	.	0.999999928	4749.22
	TNRC6A	Het	16	24834749	C	T	2.36E-04	9.29541E-05	0	0.00008721	.	1.00E+00	4749.22
	TTN	Het	2	179554626	T	TA	7.86411E-05	0	0.00E+00	.	.	1.21E-32	74772.87
Compound heterozygous missense	*TTN*	Het	2	179398591	T	C	2.36E-04	9.27E-04	0.0018	7.00E-04	7.532	1.21E-32	74772.87
	TCHH	Het	1	152084080	A	G	7.08E-04	0.001376598	0.0009	0.0006	4.628	3.87E-23	1567.52
	TCHH	Het	1	152083811	C	T	7.86411E-05	0	0	.	16.74	3.87E-23	1567.52
	ZNF599	Het	19	35250446	C	A	7.86E-05	9.29541E-05	0	1.65E-05	26.9	4.70E-07	290.08
	ZNF599	Het	19	35250438	A	T	7.86411E-05	0	0.00E+00	0.00001649	11.06	4.70E-07	290.08

Supplemental Table 3. **Whole exome sequencing data.** WES highlighting significant gene variants prioritized based upon frequency, CADD, pLI, and GDI scores accounting for the relevance of homozygosity/hemizygosity, heterozygosity, and compound heterozygosity, according to recessive and autosomal dominant models. Positions are from GRCh37/hg19.

Rank	C-score	Cluster	Program	Ligand Name	Ligand Binding	Ligand Binding Site Residues
1	0.20	10	SST	NUC	10	262,267,270,271,273,280,285,286
2	0.08	4	TMS	MG,PNY	3,1	96,100
3	0.08	4	TMS	ANP,6C6	3,1	248,250
4	0.03	1	CON			126,185,186,199,212,215,216,219,226,231,232,240,242,254,257,258,259,260,261,262,266,268,272,275,279,281,282,283,285,287,288,289,290,300,301
5	0.02	1	TMS	MG	1	253,254,257
6	0.02	1	COF	CA	1	96,166
7	0.02	1	TMS	ZN	1	166,170
8	0.02	1	TMS	CA	1	159,160
9	0.02	1	TMS	GNT	1	231,233
10	0.02	1	TMS	SE	1	215,216,219

Supplemental Table 4. **Predicted functional ligand binding sites.** COFACTOR and COACH models predicted ten potential ligand-binding sites using the described programs. These were ranked based on clustering probability, distinguishing the type of ligand and the residues that would be binding. Additionally, a binding pocket was predicted by analyzing the protein 3D structure along with its evolutionary sequence conservation.

Supplemental Materials and Methods

Whole exome sequencing and analysis

Bioinformatic filters were placed excluding benign and intronic variants, assessing for quality of sufficient read depth >20, retaining variants with a minimum variant read (vR) count >4 and minimum variant read to total read ratio >0.25 with a minor allelic frequency (MAF) < 0.005 in recessive models within the Baylor-Hopkins Center for Mendelian Genomics database, NHLBI Exome Sequencing Project Exome Variant Server (ESP 5400), 1000 Genomes, and the Exome Aggregation Consortium (ExAC) databases. Per standard pipeline analyses, data was processed by evaluating both recessive and dominant models(49). Potential recessive variants were excluded if present in the ExAC database with a homozygote or hemizygote count of 10 or greater. For autosomal dominant model analyses, variants were excluded with minor allelic frequencies >0.001 in the same databases and in the ExAC database with an allele count >5. Genes and variants respectively were filtered by their probable pathogenicity using the probability of being loss-of-function intolerant (pLI) score initially of >0.6, then depending on the individual cases of >0.8, the gene damage index (GDI) >13.84, the combined annotation-dependent depletion (CADD) >15 and the Mutation Significance Cutoffs (MSC) with gene-specific cutoffs. Additional prediction methods to determine whether a given variant is benign or deleterious such as poly-morphism phenotyping version 2 (PolyPhen-2), sorting intolerant from tolerant (SIFT), and likelihood ratio test (LRT), were considered in the final selection of plausible gene candidates.

Sanger Sequencing

Specific PCR primers for *ELF4* gene: forward 5’-CCTCCTCCTTGTTGGCTTTG-3’, reverse 5’-CTCCCTCCCCACCTCACCT-3’.

PBMC Isolation and NK cell enrichment

All human blood samples were isolated by density centrifugation over Ficoll-Paque medium, using equal parts of Ficoll, blood, and PBS. Blood diluted in PBS was layered on Ficoll, centrifuged for 20 min at 2000 rpm and subsequently collected and washed. NK and $CD34^+$ cells were enriched from PBMC using EasySep™ Human NK or CD34 cell Enrichment Kit.

T cells were expanded from PBMC by culturing them in R10 media with PHA and IL-2 for the first week and in standard R10 media stimulating the cells once weekly stimulations.

Mouse Model

MigR1 retroviral constructs carrying *ELF4* WT IRES GFP or *ELF4* T187N (c.C560A) IRES GFP were co-transfected in HEK293T cells with the retroviral construct and pEco packaging plasmid to produce retrovirus. Bone marrow (BM) cells from nonselective male and female mice were collected from 8- to 12-week-old *Elf4*$^{-/-}$ mice four days after intraperitoneal administration of fluorouracil (5-FU) 150 mg/kg. The BM cells were cultured for two days in the presence of IL-3 6 ng/mL, IL-6 10 ng/mL, and SCF 100 ng/mL, then transferred into a retronectin-coated plate and transduced with *ELF4* WT or T187N retrovirus in the presence of polybrene 8μg/mL by centrifugation at 1,290 g for 60 minutes. A second round of spinoculation was performed the following day and cultured for two days. GFP^+ BM ($ELF4^{WT}$ or $ELF4^{T187N}$) cells were purified by cell sorting. After washing with PBS, 300,000 GFP^+ cells were injected intravenously into lethally irradiated (950 rads) *Elf4*$^{-/-}$ or *Elf4*$^{+/+}$ C57BL/6J recipient mice. Three months after transplantation, blood was collected from the tail vein to assess NK reconstitution, and after confirming reconstitution, mice were euthanized to collect spleen. Mice were

stimulated by injecting Poly I:C 100µg intraperitoneally 18 to 24 hours prior to sample collection.

Cell lines and animals

C57BL/6J mice were purchased from Jackson Laboratories.

EL08.1D2 stromal cells were a gift from Dr. Jeffrey Miller, University of Minnesota, Minneapolis, MN

BLCL were produced in the lab from participants PBMC.

NK92, YTS, K562, 721.221, Raji, YAC-1, and HEK-293T cell lines were either purchased from ATCC or from long maintained and short-tandem repeat validated laboratory stocks.

Cell line Media

RPMI-1640 complete media was supplemented with 10% FBS, 2mM L-glutamine, 1mM sodium pyruvate, 1% MEM nonessential amino acid, 10mM HEPES, and 1% penicillin/streptomycin, and it was used for YTS, BLCL, K562, 721.221, Raji, and YAC-1 cell lines.

DMEM complete media supplemented with 10% FCS, 2mM L-glutamine, and 10U/mL penicillin/streptomycin and was used for culturing HEK-293T cells.

Myeolocult complete media was supplemented with 10% penicillin/streptomycin, and 100UmL^{-1} IL-2(52) was used to culture NK92 cells.

EL08.1D2 stromal cells in RPMI complete media containing 5 ng/mL IL-3 (first week only), 20 ng/mL IL-7, 10 ng/mL FLT3L, 20 ng/mL stem cell factor and 10 ng/mL IL-15 for 4 weeks

BLCL were generated by transducing PBMCs with a B95-8 EBV supernatant and cultured in RPMI complete media with 1 mg/mL cyclosporin A.

Inducible knockdown cell line production

Cells were transduced with lentivirus (reporter: turboGFP, promoter: mCMV) of three different shRNA against ELF4 (Clone Id: V3IHSMCG_ 10210331, V3IHSMCG_ 8323028, V3IHSMCG_ 9435029; Mature Antisense: TCCATATTGAGTAAGACTT, TGAAAGTAGAGGCCGCTGG, AGGTGTGCTACTGAAGTCG; Targets: ORF, ORF, 3'UTR) and one scrambled shRNA control (Cat# VSC6570) from Dharmacon SMARTvector Lentiviral shRNAs (horizon discovery).

Cytotoxicity Assay

Target cells were labeled for an hour at 37°C with 100 μCi per $1x10^6$ cells, followed by four washes in RPMI media. Targets were mixed with NK cells or T cells in a round-bottomed 96 well plate and incubated for 4 hours at 37°C. The PBMC effector cells were used in serial dilution starting at an effector to target ratio of 100:1 or 50:1 using an unstimulated control and a stimulated condition by adding 1000 U/mL of IL-2. ADCC was measured using Raji target cells incubated in the presence or absence of 20 mg/mL rituximab (anti-CD20) in a serial dilution the effector to target ratio of 100:1 or 50:1. Cytotoxicity of expanded T cells samples was measured against previously coated with CD3 antibody 1ul/mL P815 target cells. Cytotoxicity of ex vivo NK cells, NK cell lines, and isolated murine NK cells were measured in a serial dilution starting at the effector to target ratio of 10:1 or 5:1. ^{51}Cr released into supernatants from effectors and targets mix (experimental cpm), complete lysis of target cells (total cpm), and target cells without effectors were measured after centrifuging. Percentage lysis was calculated as follows:

(experimental cpm – spontaneously released cpm) / (total cpm – spontaneously released cpm) x100.

Immunofluorescence

Panel	Antibodies	Company	Clone/Catalog/Lot#		µL/reaction
In vitro development panel					
	Surface antibodies:				
	CD34	Biolegend	clone	581	15
	CD45	Beckman Coulter	clone	J33	5
	CD56	Biolegend	clone	HCD56	8
	CD16	Biolegend	clone	3G8	0.5
	CD3	Biolegend	clone	UCHT1	2
	CD127	Biolegend	clone	A019D5	25
	CD57	BD Biosciences	clone	NK-1	10
	CD117	Biolegend	clone	104D2	20
	Nkp80	Miltenyi Biotec	clone	4A4.D10	2.5
	KLRG1	Biolegend	clone	2F1/KLRG1	3
	CD294	Biolegend	clone	BM16	3
	Nkp46	Biolegend	clone	9.00E+02	4
	CD94	Biolegend	clone	DX22	10
	Lineage markers:				
	CD14	Biolegend	clone	HCD14	2
	CD19	Biolegend	clone	HIB19	2
	CD20	Biolegend	clone	2H7	2
	CD3	Biolegend	clone	SK7	2
	LIVE/DEAD Fixable Dead Cell Stains	Thermofisher			0.5
	Intracellular antibodies:				
	ELF4	Thermofisher	Catalog	PA5-63593	4
	Goat anti-rabbit secondary				0.5
	Ki67	BD Biosciences	clone	B56	10
Mice NK cell phenotype panel					
	Surface antibodies:				
	CD16	Biolegend	clone	93	2
	CD3	Biolegend	clone	17A2	4
	CD45	Biolegend	clone	30-F11	1
	Nkp46	Biolegend	clone	29A1.4	2.5
	CD27	Biolegend	clone	LG.3A10	2

	CD11b	Biolegend	clone	M1/70	3
	CD62L	Biolegend	clone	MEL-14	2
	NK1.1	Biolegend	clone	PK136	2
	CD122	Biolegend	clone	TM-β1	3
	Ly49pan	Biolegend	clone	14B11	1.25
	CD117	Biolegend	clone	2B8	1.75
	CD94	Biolegend	clone	18d3	2
	Intracellular antibodies:				
	perforin	eBioscience	clone	eBioOMAK-D	5
	Tbet	Biolegend	clone	4B10	5
	IFN-γ	Biolegend	clone	MOB-47	5
	Eomes	eBioscience	clone	Dan11mag	5
	GFP	Biolegend	clone	FM264G	5
NK cell line phenotype panel					
	Surface:				
	LIVE/DEAD Fixable Dead Cell Stains	Thermofisher			0.5
	Intracellular:				
	ELF4	Thermofisher	Catalog	PA5-63593	4
	Goat anti-rabbit secondary				0.5
	perforin BD48	Biolegend	clone	BD48	5
	perforin δG9	Biolegend	clone	δG9	5
	granzyme B	Biolegend	clone	BG11	5
ELF4 phenotype panel					
	Intracellular:				
	ELF4	Thermofisher	Catalog	PA5-63593	4
	Goat anti-rabbit secondary				0.5
	BrdU	BD Biosciences	Catalog	559619	1
	DAPI				
T cell phenotype panel					
	Surface antibodies:				
	LIVE/DEAD Fixable Dead Cell Stains	Thermofisher			1
	CD3 (S4.1)	Invitrogen	Lot #	2264236	2
	CD45RA	BD Biosciences	Lot #	9351398	2
	CD45RO	Biolegend	Lot #	B283201	2
	CD57	Biolegend	Lot #	B329649	2
	CD8a	Biolegend	Lot #	B332858	1
	CD4	Biolegend	Lot #	B311736	1
	Intracellular antibodies:				

	perforin δG9	Biolegend	clone	δG9	3
	granzyme B	Biolegend	clone	BG11	2

Imaging Flow Cytometry

An Amnis MKII imaging flow cytometer was utilized with voltages calibrated with beads and single stained cells before the initial acquisition; at this time, compensation tubes were also acquired (in the absence of brightfield). In the INSPIRE acquisition software suite, the following parameters were used to select the count number of cells to stop the acquisition collecting all events; the appropriate size (Area_M01 >50) and circularity (Aspect Ratio_01 >.6) based on the brightfield parameter, followed by the in-focus cells (Gradient RMS_M01 >60), and selecting cells that were not overexposed in any channels used (Raw Max Pixel_MC_Ch <4e3). Uncompensated gates of the specified markers were used when needed (Intensity_MC_Ch) to select cells from specific populations. At least 1000 cells were acquired per sample and 500 events for compensation controls.

The data was analyzed as follows using the IDEAS software. A compensation matrix file (.ctm) was created from raw image file (.rif) samples acquired with no bright field using both single stained cells and single stained compensation beads. Selecting all sample plots of Object number vs. intensity of all channels were created (Intensity_MC_Ch), from which the cluster of object numbers with the highest intensity were gated as the populations for each channel, eliminating outliers to reduce error below 1% on any coefficient numbers. All samples rif files were batch processed with the selected compensation matrix file creating a new compensated image file (.cif). Using one of the samples' cif files to create an analysis template file (.ast) the following basic analysis was applied: gate cells selecting for the focused cells (Gradient RMS_M01), the appropriate size (Area_M01), circularity (Aspect Ratio_01), using only cells that were not overexposed in all used channels (Raw Max Pixel_MC_Ch), and live cells based

on DAPI (Intensity_MC_Ch). Subsequently, Nuclear localization was calculated by creating a cellular mask using the fill function on the brightfield channel and a nuclear mask using the erode function or adaptive erode function with the nuclear dye channel; then, multiple features were made by adding the new masks and the intensity of ELF4, at last, a new combined feature was created (Intensity_Nuclear Mask_Ch ELF4/Intensity_Cellular Mask_Ch ELF4*100). The geometric mean on the new feature Nuclear Localization defines the average Nuclear localization of the selected sample. Sample cif data files were batch processed with an analysis template creating a new data analyzed file (.daf).

To create figure graphs, samples were merged into a single analysis file. All samples were merged using a reduced cif file of live-focused cells, and the analysis template was applied. In the merged daf file, gates were applied to all populations to select graphs for figures and export statistics.

Transfection

Plasmids had a pCDH-CMV-MCS-EF1a-puro backbone and expressed *ELF4* NM_001127197.2 mRNA WT or T187N variant (c.C560A p.T187N). All constructs were generated by Epoch Life Science custom cloning services.

Luciferase Assay

Constructs in the pIRES eGFP expression vector encoding ELF4 WT or ELF4 T187N variant were generated by Epoch Life Science custom cloning services. LightSwitch™ Promoter Reporter Vectors MDM2 (product ID: S704939) and Perforin (product ID: S719322) were co-transfected in HEK293T for LightSwitch Luciferase Assay.

ChIP-qPCR

Samples were cross-linked with media supplemented with 1% formaldehyde

and gently rotated at room temperature for 10 minutes. The reaction was quenched by gently rotating for 5 minutes with media supplemented with glycine and rinsed twice with cold PBS. Cells were centrifuged at 500g for 5 minutes at 4°C, and the pellet was resuspended in $2x10^6$ cells per mL lysis buffer. After a 15-minute incubation on ice, the supernatant containing the cytoplasmic fraction was carefully aspirated. The pellet was resuspended in 100µL of nuclear lysis buffer, incubated for 15 min on ice, and sonicated following instructions provided by Covaris. Processed supernatants were collected after centrifugation at top speed for 15 minutes and confirmed sonication efficiency by treating a 5µL aliquot with 0.5µL of Proteinase K and confirming the size of the fragments was between 200 to 500 bp. Beads were equilibrated by incubating with 25µL of trap-myc antibody and binding control in dilution buffer and subsequently removing supernatant after magnetic separation. A100µL of the sheared chromatin diluted in 900µL of dilution buffer was pre-cleared by adding 30µL of binding control (chromotek bmab-20) and incubating for 2 hours at 4°C on a rotator. After pre-clearing, 4% of chromatin was stored as input at −20C°, and the remaining chromatin was transferred to the equilibrated anti-myc nanobody (chromotek ytma-20) or binding control beads and incubated overnight at 4°C while rotating to immunoprecipitate the myc- tagged ELF4 protein. Samples were separated magnetically and subsequently washed with the following cold buffers: once with wash buffer 1 and wash buffer 2, then again twice with wash buffer 1, then once with wash buffer 3 and TE buffer. Input and IP samples were decrosslinked in parallel by resuspending in 100µL of Elution buffer, incubating 10 minutes at 65°C, transferring eluate to a new tube after magnetic separation, and adding NaCl for a final concentration of 0.2M and RNAse for a final concentration of 10µg/mL for overnight incubation. The samples were then incubated with

EDTA for a final concentration of 5mM and proteinase K for a final concentration of 0.1mg/mL to proceed with the DNA purification and subsequent quantitative real-time PCR.

Reagents and Buffers:

- Glycine supplemented media (glycine 125mM)
- Lysis buffer (Pipes [pH 8.0] 4°C 5mM, NP-40 0.5%, KCl 85mM, IP 1X, HALT 1X, PMSF 1mM, and water)
- Nuclear lysis buffer (Tris-HCl [pH 8.0] 50mM, EDTA 10mM, SDS 0.8%, IP 1X, HALT 1X, PMSF 1mM, and water)
- Dilution buffer (Tris-HCl [pH 8.0] 10mM, EDTA 0.5mM, TritonX 1%, IP 1X, NaCl 140mM, PMSF 1mM, and water)
- Wash buffer 1 (Tris-HCl [pH8] 20mM, NaCl 150mM, TritonX 1%, EDTA 2mM, SDS 0.1% and water)
- Wash buffer 2 (Tris-HCl [pH8] 20mM, NaCl 500mM, TritonX 1%, EDTA 2mM, SDS 0.1% and water)
- Wash buffer 3 (Tris-HCl [pH8] 10mM, LiCl 250mM, NP-40 1%, Na-deoxycholate 0.5%, EDTA 1mM, and water)
- TE buffer (Tris-HCl [pH8] 10mM, 1mM, SDS 0.1% and water)
- Elution buffer (Tris-HCl [pH8] 50mM, EDTA 1mM, SDS 0.1%, and water)
- Perforin promoter (assay ID: ARFVMY9, AAACTGAGGCACAGTGAGGTGAAG)
- MDM2 promoter (assay ID: ARGZGJ6, CCTGCCTTAAGTGCTATTTTTAATC)

Cell Fractionation and Salt Extraction

20 million cells were collected per condition, washed, and divided into 4 tubes. To collect the total cell lysate: one tube was treated with 150μL of NP-40 Lysis buffer, incubated for 20

minutes on ice, and centrifuged at top speed for 20 minutes to collect the supernatant. The others were treated with 150µL of CEBN Buffer, incubated for 3 minutes, and centrifuged at 5,000 rpm for 5 minutes to collect the cytoplasmic fraction. The pellets of the remaining samples were washed with 150µL of CEB Buffer. Subsequently, one tube was separated and treated with NP-40 lysis buffer to collect the total nuclear fraction. The rest of the samples were treated with 50µL of nuclear extraction buffer, incubated for 3 minutes on ice, centrifuged at 13,000rpm for 3 minutes, and the nuclear soluble fraction from the supernatant was collected. Subsequently, one tube was separated and treated with NP-40 lysis buffer to collect the total chromatin-bound fraction. The remaining sample was treated with 50µL of salt extraction buffer, incubated for 3 minutes on ice, centrifuged at 13,000rpm for 3 minutes, and the supernatant collected. Samples were treated starting with the lowest NaCl concentration at 0.03M and repeated with the incrementing concentrations of the salt extraction buffer serial dilutions.

Buffers:

- Lysis buffer (Tris-HCL [pH 7.5] 35mM, NP-40 1%, Glycerol 5%, NaCl 150mM, EDTA 1mM, and H2O)
- CEBN Buffer (Hepes [pH 7.8] 10mM, KCL 10mM, MgCl2 1.5 mM, Glycerol 10%, HALT 1X, Sucrose 0.34M, NP-40 0.2%, and water)
- CEB Buffer (HEPES [pH 7.8] 10mM, KCL 10mM, MgCl2 1.5mM, Glycerol 10%, HALT 1X, Sucrose 0.34M, and water)
- Nuclear extraction buffer (Tris [pH 8.0] 50mM, NP-40 0.05%, HALT 1X and water)
- Salt extraction buffer (serial dilution of 0.5M of NaCl in nuclear extraction buffer)

Western Blot

Cells were lysed at a concentration of 20 million cells per mL by adding prepared buffer, incubating on ice for 20 minutes, and centrifuging at top speed for 20 minutes to collect supernatant. ELF4 antibody was used at a 1/2500 dilution. Blots were imaged and quantified using Li-Cor's Odyssey imaging system.

Buffer:

RIPA buffer was prepared to add 1μL of HALT per mL of RIPA.

Protein Modeling and Analysis

The top structures generated through the I-TASSER predicted full-length 3D-model server that was used had a TM score of 0.60 and a C-score of -0.9 (range [-5,2]). The software template-based modeling, along with its threading algorithm, evaluates the predicted structure based on the quality of the fit to previously crystallized structures and allows for the use of some Ab initio structure prediction. This is important for ELF4 because domains like the ETS domain have been crystallized in other proteins, but other domains have not been crystallized and have poor homology.

Manufacturer and Catalog numbers

Gentra PureGene Blood Kit (QIAGEN Cat:158445).

4Peaks software (Nucleobytes)

Ficoll-Paque (Amersham Fisher Scientific, Cat:17144002)

EasySep™ Human NK Cell Enrichment Kit (StemCell Technologies, Cat:19055)

Rosettesep NK cell enrichment (Stemcell Technologies)

All cytokines (Peprotech)

BrdU Flow Kit (BD biosciences, Cat:552598)

Dharmacon SMARTvector Lentiviral shRNAs (horizon discovery).

Scrambled shRNA control (horizon discovery, Cat: VSC6570)

TransDux Max (Systems Biosciences, Cat: LV860A-1)

Lumaplate and TopCount XL gamma detector (Perkin-Elmer)

Cytofix/Cytoperm buffer (BD Biosciences, Cat: 554714)

Foxp3/Transcription Factor Staining Buffer Kit (TONBO Biosciences, Cat: TNB-0607-KIT)

FuGene (Promega, Cat: E2691)

LightSwitch™ Promoter Reporter Vectors (SwitchGear Genomics)

LightSwitch Luciferase Assay Reagent (Active Motif)

ELF4 (Sigma, Cat: AV38028)

Odyssey imaging system (Li-Cor)

Prism 9.1.0 (GraphPad Software)

Relevant websites utilized and referred to:

Whole exome sequencing and analysis

RefSeq, http://www.ncbi.nlm.nih.gov/RefSeq

UCSC Genome Browser, https://genome.ucsc.edu/

Baylor Genetics Laboratory, http://bmgl.com/

NHLBI Exome Sequencing Project (ESP) Exome Variant Server,

http://evs.gs.washington.edu/EVS/

1000 Genomes, http://www.internationalgenome.org/

GnomAD, http://gnomad.broadinstitute.org/

DECIPHER, https://decipher.sanger.ac.uk/

Combined Annotation-Dependent Depletion (v.1.0), http://cadd.gs.washington.edu/home

Human Gene Damage Index (GDI), http://lab.rockefeller.edu/casanova/GDI

Mutation Significance Cutoffs (MSC), https://lab.rockefeller.edu/casanova/MSC

SIFT, http://sift.jcvi.org

PolyPhen-2, http://genetics.bwh.harvard.edu/pph2/

MutationTaster, http://www.mutationtaster.org/

FATHMM, http://fathmm.biocompute.org.uk/fathmmMKL.htm

HGMD Professional, http://www.biobase-international.com/product/hgmd

ClinVar, https://www.ncbi.nlm.nih.gov/clinvar/

OMIM, http://www.omim.org/

Cufflinks, http://cufflinks.cbcb.umd.edu

Clinical Genomic Database, http://research.nhgri.nih.gov/CGD/

GenBank, http://www.ncbi.nlm.nih.gov/genbank/

GeneMatcher, https://genematcher.org/

HTseq, http://www-huber.embl.de

Integrative Genomics Viewer (IGV), http://software.broadinstitute.org/software/igv

Sanger Sequencing

Primer3, http://biotools.umassmed.edu/bioapps/primer3_www.cgi

Protein Modeling and Analysis

I-Tasser, https://zhanglab.dcmb.med.umich.edu/I-TASSER/

Ligplot, https://www.ebi.ac.uk/thornton-srv/software/LigPlus/

UCSF Chimera, https://www.rbvi.ucsf.edu/chimera/

Data Availability

https://doi.org/10.5061/dryad.5tb2rbp3x
